# Quantum enhanced multiple-phase estimation with multi-mode *N*00*N* states

**DOI:** 10.1038/s41467-021-25451-4

**Published:** 2021-09-01

**Authors:** Seongjin Hong, Junaid ur Rehman, Yong-Su Kim, Young-Wook Cho, Seung-Woo Lee, Hojoong Jung, Sung Moon, Sang-Wook Han, Hyang-Tag Lim

**Affiliations:** 1 Center for Quantum Information, Korea Institute of Science and Technology (KIST), Seoul, Korea; 2 Department of Electronics and Information Convergence Engineering, Kyung Hee University, Yongin, Korea; 3 Division of Nano and Information Technology, KIST School, Korea University of Science and Technology, Seoul, Korea; 4 Department of Physics, Yonsei University, Seoul, Korea

**Keywords:** Quantum optics, Quantum information, Quantum metrology

## Abstract

Quantum metrology can achieve enhanced sensitivity for estimating unknown parameters beyond the standard quantum limit. Recently, multiple-phase estimation exploiting quantum resources has attracted intensive interest for its applications in quantum imaging and sensor networks. For multiple-phase estimation, the amount of enhanced sensitivity is dependent on quantum probe states, and multi-mode *N*00*N* states are known to be a key resource for this. However, its experimental demonstration has been missing so far since generating such states is highly challenging. Here, we report generation of multi-mode *N*00*N* states and experimental demonstration of quantum enhanced multiple-phase estimation using the multi-mode *N*00*N* states. In particular, we show that the quantum Cramer-Rao bound can be saturated using our two-photon four-mode *N*00*N* state and measurement scheme using a 4 × 4 multi-mode beam splitter. Our multiple-phase estimation strategy provides a faithful platform to investigate multiple parameter estimation scenarios.

## Abstract (web summary)

N00N states are a key resource in quantum metrology, but the use of their multi-mode extension for multiparameter estimation has been elusive so far. Here, the authors use multi-mode N00N states - with N=2 photons in 4 modes - for multiple-phase estimation saturating the quantum Cramer-Rao bound.

## Introduction

Quantum metrology has attracted intensive interest in recent years, as it allows us to estimate an unknown parameter with enhanced sensitivity over classical approaches. Developments are now directed towards various applications such as microscopy^1–5^, biological imaging^6–10^ and sensor network^11–13^. In such practical applications, quantum metrology is naturally extended to multiple parameter scenario, since the number of parameters that affect a physical process is generally more than one. For estimating multiple parameters, simultaneous estimation is desirable as it can achieve higher precision than an individual estimation of each parameter using the same amount of resources^14,15^. Furthermore, if a physical system of interest has time dynamics, estimation of multiple parameters has to be done simultaneously. However, unlike single parameter estimation, it is non-trivial to optimize multiple parameter estimation to achieve the maximum sensitivity. Various strategies have been thus proposed to enhance the sensitivity by optimizing either the probe states or the measurement scheme^14–22^. For example, quantum strategies exploiting various quantum probe states such as Greenberger–Horne–Zeilinger states^23^, single-photon Fock states^24^, squeezed states^12,25–28^, Holland-Bernett states^29–31^, and *N*00*N* states^14,32,33^ have been extensively studied.

In particular, *N*00*N* states have been outstanding to investigate the fundamental quantum limit of quantum metrology given by the Heisenberg uncertainty principle with a fixed number of particles^34^. In a single-phase estimation scheme, *N*00*N* states can saturate the Heisenberg limit thanks to its largest number variance between the two modes^34^. Enhanced sensitivity beyond the standard quantum limit has been experimentally demonstrated with *N*00*N* states^35,36^. Recently, the concept of *N*00*N* states has been extended to generalized multi-mode *N*00*N* states to investigate the quantum enhancement in multiple-phase estimation^14^. Moreover, for multiple-phase estimation with limited resources, it has been known that multi-mode *N*00*N* states allow achieving the enhanced sensitivity outperforming the other quantum probe states or classical strategies for a multi-mode interferometer^14,15,32^. However, since generation of such multi-mode quantum probe states is challenging, experimental demonstrations of multiple parameter quantum metrology have been limited to utilizing quantum states other than multi-mode *N*00*N* states^12,15,23,24,27,29^.

In this work, we experimentally demonstrate quantum enhanced multiple-phase estimation using a multi-mode *N*00*N* state with photon number *N* = 2 and mode number *m* = 4. To this end, we propose a scheme for generating multi-mode *N*00*N* states. At first, we observe that the measured interference fringes exhibit phase super-resolution as a function of phase differences *φ*_*i*_ (*i* = 1, 2, 3) of the generated 4-mode 2002 state with a measurement scheme using a 4 × 4 multi-mode beam splitter, so-called a quarter^37,38^. Then, we demonstrate quantum enhanced phase sensitivity of the 4-mode 2002 state by analyzing the Cramer-Rao bound (CRB) and the quantum Cramer-Rao bound (QCRB)^14,16,17,39^. Moreover, we show that the measured sensitivity is better than a coherent state $|\alpha \rangle$, which is a classical probe state, as well as another quantum probe state prepared by single-photon Fock states^24^. Our results can motivate an investigation into the quantum strategies using multi-mode and multi-particle entanglement to develop the quantum enhanced multiple parameter metrology.

## Results

### Multiple-phase estimation scenario

Let us begin by introducing the system model for multiple-phase estimation schemes with multi-mode *N*00*N* states defined as

1 $$\left|{\psi }_{m}^{N}\right\rangle =\frac{1}{\sqrt{m}}(\left|N0\cdots 0\right\rangle +\left|0N0\cdots 0\right\rangle +\cdots +\left|0\cdots 0N\right\rangle ),$$

where *N* is the number of photons, and they are distributed along *m* modes^14,32,33^. The multi-mode *N*00*N* state is a coherent superposition of all possibilities where *N* photons in one mode and none in any of the other *m* − 1 modes^34^. Then, we theoretically analyze the sensing scheme of *d* = *m* − 1 unknown multiple phases and the fixed photon number *N* = 2 with different probe states. A quantum probe state undergoes individual phase shifts ***φ*** = {*φ*_1_, *φ*_2_, ⋯ , *φ*_*d*_} and the phase shifted state is combined using an *m* × *m* multi-mode beam splitter and then detected by photon number-resolving detectors (PNRDs) at each mode. Here, the goal is to minimize the total uncertainty of the phase estimation governed by the CRB and the QCRB. The lower bound of the sum of the variance of each phase estimation given by the CRB and the QCRB is^14,16,17,39^

2 $$\mathop{\sum }\limits_{i=1}^{d}| {{\Delta }}{\varphi }_{i}{| }^{2}\ge \frac{\,{{\mbox{Tr}}}\,[{{{{{{{{\bf{F}}}}}}}}}_{C}^{-1}({{{{{{{\boldsymbol{\varphi }}}}}}}})]}{\mu }\ge \frac{\,{{\mbox{Tr}}}\,[{{{{{{{{\bf{F}}}}}}}}}_{Q}^{-1}({{{{{{{\boldsymbol{\varphi }}}}}}}})]}{\mu },$$

where **F**_*C*_(***φ***) is the classical Fisher information matrix (CFIM), **F**_*Q*_(***φ***) is the quantum Fisher information matrix (QFIM), and *μ* is the number of measurements. Note that **F**^−1^ refers to the inverse of the Fisher information matrix **F**. The first and second inequalities are direct consequences of the CRB and the QCRB, respectively. Note that in our experiments, the CRB can always be saturated, asymptotically in *μ*, by using maximum likelihood estimator, and the mean of phases are expected to converge to the true values^17,39^.

**Fig. 1:**
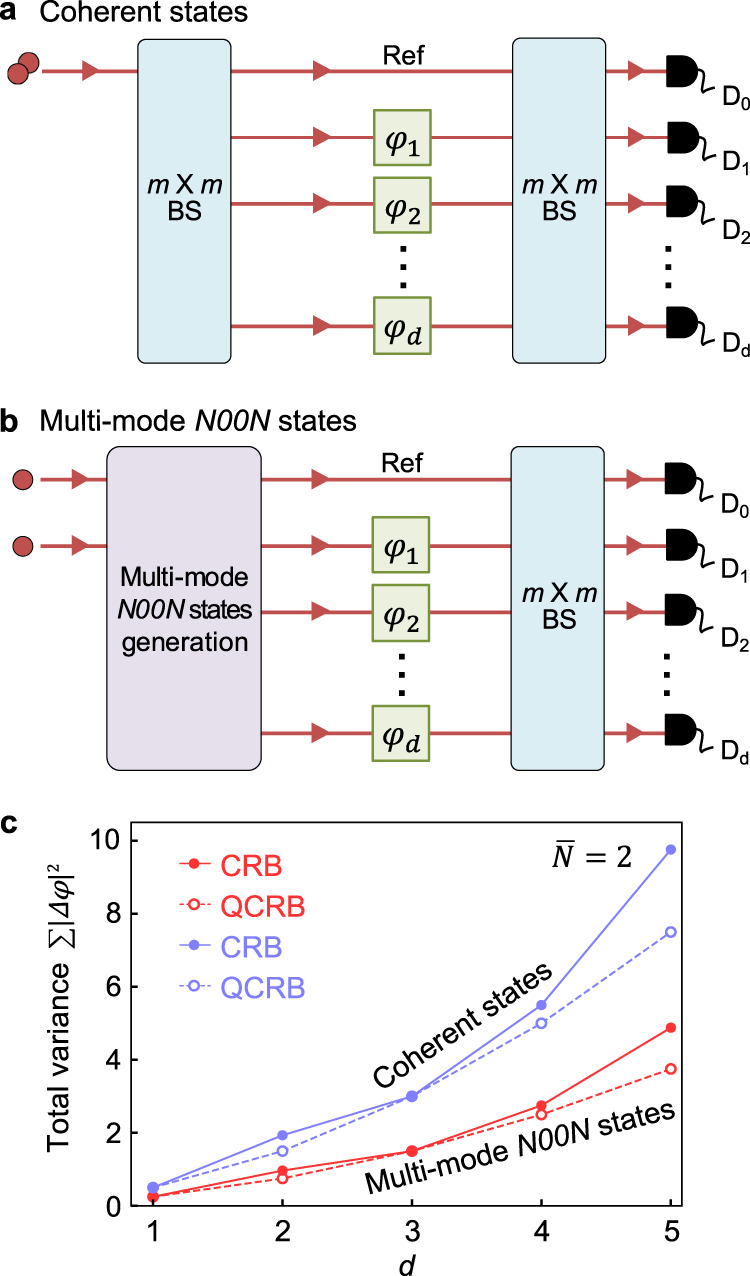
Quantum enhanced multiple-phase estimation scheme. The goal is to estimate the *d* multiple phases with phase shifts *φ*_1_, …, *φ*_*d*_ while minimizing the total uncertainty of phase estimation governed by the CRB and the QCRB. To estimate the multiple phases, coherent states with $\overline{N}=2$ (**a**) and (*d* + 1)-mode *N*00*N* states with *N* = 2 (**b**) are used for probe states. **c** The total variance of multiple-phase estimation obtained by the CRB and the QCRB as a function of the number of unknown phases *d* with two probe states. It clearly shows that multi-mode *N*00*N* states always have better sensitivity compared to coherent states.

We theoretically analyze the CRB and the QCRB of *d* multiple-phase estimation scheme with two different probe states. The first probe state we consider is a classical state prepared by injecting a coherent state $\left|\alpha \right\rangle$ with an average photon number $\overline{N}=2$ into one of the input ports of an *m* × *m* multi-mode beam splitter (Fig. 1a), and the other probe state is the two-photon *m*-mode *N*00*N* state (Fig. 1b). The measurement scheme is identical for two probe states. Each mode of the probe states is combined at another *m* × *m* multi-mode beam splitter after undergoing phase shifts, and then measured by PNRDs. We provide the CRB and the QCRB values of the total variance $\sum {\left|{{\Delta }}\varphi \right|}^{2}$ for two probe states of coherent states and multi-mode *N*00*N* states with various number of phases *d* in Fig. 1c. Note that multi-mode *N*00*N* states always have lower phase uncertainty than coherent states, i.e., classical probe states.

### Generation of the 4-mode 2002 state

**Fig. 2:**
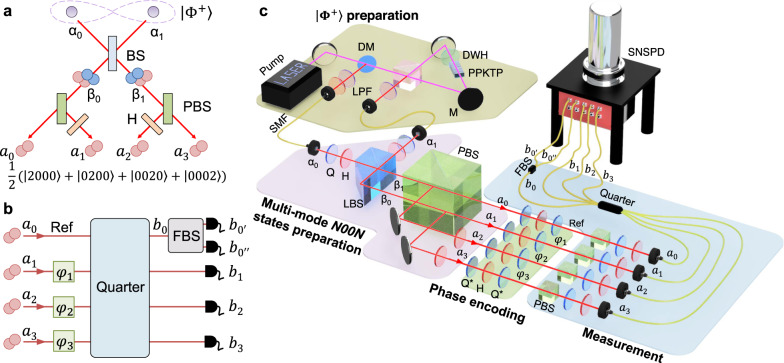
Schematic of the experimental setup. **a** A simplified scheme of preparing 4-mode 2002 state $\left|{\psi }_{4}^{2}\right\rangle$ from $\left|{{{\Phi }}}^{+}\right\rangle$ using the Hong-Ou-Mandel interference effect^48^, see Eq. (3). **b** The prepared $\left|{\psi }_{4}^{2}\right\rangle$ undergoes the phase shift *φ*_*i*_ (*i* = 1, 2, 3) in each mode. After the phase encoding, a quarter is used for combining four modes of the probe state, and then the two-photon output states are detected using single-photon detectors. In particular, one of the output modes of quarter (*b*_0_) is divided into two modes using a FBS for two-photon detection in that mode. **c** Experimental setup for generating $\left|{\psi }_{4}^{2}\right\rangle$ state and performing quantum enhanced multiple-phase estimation. See Methods for detailed information on $\left|{\psi }_{4}^{2}\right\rangle$ state generation. The prepared probe state $\left|{\psi }_{4}^{2}\right\rangle$ undergoes phase encoding using a set of Q*-H-Q* located in each mode. Then, after the quarter and the FBS, the two-photon coincidence counts are measured using SNSPDs. BS: beam splitter; PBS: polarizing beam splitter; DM: dichroic mirror; DWH: dual wavelength half waveplate; PPKTP: periodically poled KTiOPO; M: mirror; LPF: long pass filter; SMF: single mode fiber; Q: quarter waveplate; H: half waveplate; LBS: lateral beam splitter; Q*: quater waveplate with an optic axis of 45^∘^; FBS: 50/50 fiber beam splitter; SNSPD: superconducting nanowire single-photon detector.

The conceptual diagram of our proposed scheme for preparing the multi-mode *N*00*N* state with *N* = 2 and *m* = 4 is shown in Fig. 2a. The generation process of 4-mode 2002 state $|{\psi }_{4}^{2}\rangle$ is the following:

3 $${|}{{{\Phi }}}^{+}\rangle = \frac{1}{\sqrt{2}}\left(|{1}_{{\alpha }_{0}}^{H}{1}_{{\alpha }_{1}}^{H}\rangle +|{1}_{{\alpha }_{0}}^{V}{1}_{{\alpha }_{1}}^{V}\rangle \right)\\ \mathop{\to }\limits^{{{{{{{{\rm{BS}}}}}}}}} \frac{1}{2}\left(|{2}_{{\beta }_{0}}^{H}{0}_{{\beta }_{1}}\rangle +|{0}_{{\beta }_{0}}{2}_{{\beta }_{1}}^{H}\rangle +|{2}_{{\beta }_{0}}^{V}{0}_{{\beta }_{1}}\rangle +|{0}_{{\beta }_{0}}{2}_{{\beta }_{1}}^{V}\rangle \right)\\ \mathop{\to }\limits^{{{{{{{{\rm{PBS}}}}}}}},{{{{{{{\rm{HWP}}}}}}}}} \frac{1}{2}\left(|{2}_{{a}_{0}}^{H}{0}_{{a}_{1}}{0}_{{a}_{2}}{0}_{{a}_{3}}\rangle +|{0}_{{a}_{0}}{2}_{{a}_{1}}^{H}{0}_{{a}_{2}}{0}_{{a}_{3}}\rangle \right.\\ +\,\left.|{0}_{{a}_{0}}{0}_{{a}_{1}}{2}_{{a}_{2}}^{H}{0}_{{a}_{3}}\rangle +|{0}_{{a}_{0}}{0}_{{a}_{1}}{0}_{{a}_{2}}{2}_{{a}_{3}}^{H}\rangle \right).$$

Here, ${|}{{{\Phi }}}^{+}\rangle =\frac{1}{\sqrt{2}}(|{1}_{{\alpha }_{0}}^{H}{1}_{{\alpha }_{1}}^{H}\rangle +|{1}_{{\alpha }_{0}}^{V}{1}_{{\alpha }_{1}}^{V}\rangle )$ is the triplet Bell state where, for example, $|{1}_{{\alpha }_{0}}^{H}\rangle$ denotes the horizontally polarized single-photon state in the mode *α*_0_. Our scheme can generate $\left|{\psi }_{4}^{2}\right\rangle$ with a unity conversion probability from the pre-selected initial $\left|{{{\Phi }}}^{+}\right\rangle$ state into $\left|{\psi }_{4}^{2}\right\rangle$, thus two-photons always move together, see Methods for the detailed information. Then, prepared $\left|{\psi }_{4}^{2}\right\rangle$ undergoes phase encoding *φ*_*i*_ (*i* = 1, 2, 3) and it is combined by a quarter as shown in Fig. 2b. The output states are measured by single-photon detectors located at each mode. Note that one output port of the quarter is split into two (${b}_{0^{\prime} }$ and *b*_0*″*_) using a 50/50 fiber beam splitter to implement a pseudo-PNRD. Post-selected two-photon coincidence counts of both output port ${b}_{0^{\prime} }$ and *b*_0*″*_ correspond to the two-photon counting at *b*_0_ with success probability of 1/2. The schematic of experimental setup is shown in Fig. 2c.

**Fig. 3:**
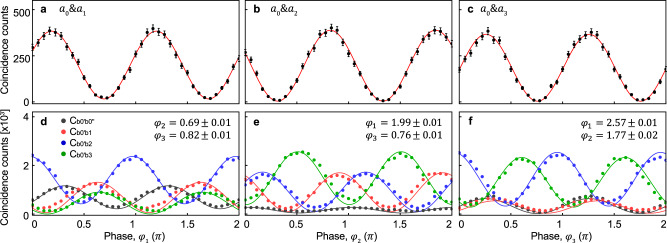
Measured interference fringes for the $\left|{\psi }_{4}^{2}\right\rangle$ probe state. **a**–**c** Among four modes of $\left|{\psi }_{4}^{2}\right\rangle$, two input modes are blocked while the reference mode *a*_0_ and the one of the other modes *a*_*i*_ with *i* = 1, 2, 3 are used. Two-photon coincidence counts ${{{\mbox{C}}}}_{{b}_{0^{\prime} }{b}_{0^{\prime\prime} }}$ between two modes $b_{0}^{\prime}$ and $b_{0}^{{\prime}{\prime}}$ are measured with varying the phase encoding *φ*_*i*_. Experimental data are fitted with a sinusoidal function, which has two times faster modulation period with respect to the single-photon input case and has a good agreement with the visibility *V* = 0.920 (**a**), 0.980 (**b**), and 0.979 (**c**), respectively. **d**–**f** Interference fringes obtained by two-photon coincidences of ${{{\mbox{C}}}}_{{b}_{0^{\prime} }{b}_{0^{\prime\prime} }}$, ${{{\mbox{C}}}}_{{b}_{0^{\prime} }{b}_{1}}$, ${{{\mbox{C}}}}_{{b}_{0^{\prime} }{b}_{2}}$, and ${{{\mbox{C}}}}_{{b}_{0^{\prime} }{b}_{3}}$ (black, red, blue, and green, respectively) with scanning one of *φ*_*i*_(*i* = 1, 2, 3) when all of the input modes of $\left|{\psi }_{4}^{2}\right\rangle$ are used. Solid lines represent theoretical calculations based on an ideal $\left|{\psi }_{4}^{2}\right\rangle$ input state and an experimentally reconstructed quarter transition matrix. All error bars represent one standard deviation.

As a first step to verify the generation of $\left|{\psi }_{4}^{2}\right\rangle$, we investigate the coherence among all four modes of $\left|{\psi }_{4}^{2}\right\rangle$ by observing the interference fringes between the reference mode (*a*_0_) and one of the other modes (*a*_*i*_). We measure the two-photon coincidence counts on ${b}_{0^{\prime} }$ and *b*_0*″*_, ${{{\mbox{C}}}}_{b0^{\prime} b0^{\prime\prime} }$, of two input modes while we block the other two input modes. The phase shift *φ*_*i*_ is realized by adjusting the optic axis angle *θ*_*i*_ of the half waveplate (HWP) located between two quarter waveplates (QWPs). Note that *φ*_*i*_ = 2*θ*_*i*_. As shown in Fig. 3a–c, results of ${{{\mbox{C}}}}_{b0^{\prime} b0^{\prime\prime} }$ clearly reveal two times faster sinusoidal modulations compared to the single-photon input case due to the *λ*/2 photonic de Broglie wavelength of two-photon *N*00*N* states^40–42^.

Then, in order to directly confirm the generation of $\left|{\psi }_{4}^{2}\right\rangle$, we measure two-photon probability distributions for the output states when we use all of the input modes *a*_0_, *a*_1_, *a*_2_, and *a*_3_ of $\left|{\psi }_{4}^{2}\right\rangle$ while we vary one of the phase encoding *φ*_*i*_. The experimental results of the interference fringes from all of the modes are shown in Fig. 3d–f, and they are compared with the theoretical calculations. We obtain the theoretical predictions of ${{{\mbox{C}}}}_{{b}_{0}{b}_{0}}$, ${{{\mbox{C}}}}_{{b}_{0}{b}_{1}}$, ${{{\mbox{C}}}}_{{b}_{0}{b}_{2}}$, and ${{{\mbox{C}}}}_{{b}_{0}{b}_{3}}$ based on the ideal $\left|{\psi }_{4}^{2}\right\rangle$ and the experimentally reconstructed quarter transition matrix. Then we experimentally measured post-selected coincidence counts of C_*i*_ for $i={b}_{0^{\prime} }{b}_{0^{\prime\prime} },{b}_{0^{\prime} }{b}_{1},{b}_{0^{\prime} }{b}_{2}$ and ${b}_{0^{\prime} }{b}_{3}$. See Supplementary Note 1 for the detailed information on a quarter transition matrix and theoretical calculations. The results of Fig. 3d–f shows that the experimentally obtained interference fringes without normalization are very well-matched to the theoretical calculations, thus we can confirm that the prepared input state is $\left|{\psi }_{4}^{2}\right\rangle$.

### Experimental multiple-phase estimation

Then, we investigate the sensitivity bound of multiple-phase estimation using our prepared $\left|{\psi }_{4}^{2}\right\rangle$ as a probe state. At first, we theoretically calculate the CRB with an ideal $\left|{\psi }_{4}^{2}\right\rangle$ and an ideal quarter when the CRB saturates the QCRB. We obtain the theoretical two-photon detection probability set of {*P*_*l*_(***φ***)} (*l* = 0, 1, . . . , 9) with a set of projectors $\{{\hat{{{\Pi }}}}_{l}\}=\left\{\left|2000\right\rangle \right.\left\langle 2000\right|$, $\left|0200\right\rangle \left\langle 0200\right|$, $\left|0020\right\rangle \left\langle 0020\right|$, $\left|0002\right\rangle \left\langle 0002\right|$, $\left|1100\right\rangle \left\langle 1100\right|$, $\left|0011\right\rangle \left\langle 0011\right|$, $\left|1010\right\rangle \left\langle 1010\right|$, $\left|0101\right\rangle \left\langle 0101\right|$, $\left|1001\right\rangle \left\langle 1001\right|$, $\left|0110\right\rangle \left.\left\langle 0110\right|\right\}$ satisfying the normalizing condition ∑_*l*_*P*_*l*_(***φ***) = 1. Note that *P*_0_ = *P*_1_ = *P*_2_ = *P*_3_, *P*_4_ = *P*_5_, *P*_6_ = *P*_7_, and *P*_8_ = *P*_9_ for all ***φ*** (See Methods). Then CFIM is given by

4 $${F}_{C(j,k)}=\mathop{\sum }\limits_{l=0}^{9}\frac{1}{{P}_{l}({{{{{{{\boldsymbol{\varphi }}}}}}}})}\left(\frac{\partial {P}_{l}({{{{{{{\boldsymbol{\varphi }}}}}}}})}{\partial {\varphi }_{j}}\right)\left(\frac{\partial {P}_{l}({{{{{{{\boldsymbol{\varphi }}}}}}}})}{\partial {\varphi }_{k}}\right),$$

where *j* and *k* can be 1, 2, and 3. The minimum value of the CRB is obtained to be $$\,{{\mbox{Tr}}}\,[{{{{{{{{\bf{F}}}}}}}}}_{C}^{-1}({{{{{{{\boldsymbol{\varphi }}}}}}}})]$$ = 1.5 where *φ*_1_ ≃ *π*/2, *φ*_2_ = 0, and *φ*_3_ = *π*/2, and it saturates the QCRB = 1.5. In order to experimentally estimate the CRB value, we obtained interference fringes by scanning *φ*_1_ near the point where we expected both the CRB and the QCRB to be saturated (*φ*_2_ = −0.07*π* and *φ*_3_ = 0.52*π*) for the prepared $${\left|{\psi }_{4}^{2}\right\rangle }_{\exp }$$ probe state (See Methods). Two-photon detection probabilities $${P}_{{b}_{0}{b}_{0}}^{\,{{\mbox{m}}}\,}$$, $${P}_{{b}_{0}{b}_{1}}^{\,{{\mbox{m}}}\,}$$, $${P}_{{b}_{0}{b}_{2}}^{\,{{\mbox{m}}}\,}$$ and $${P}_{{b}_{0}{b}_{3}}^{\,{{\mbox{m}}}\,}$$ are then obtained from the measured post-selected coincidence counts with *μ* ≃ 8, 144, C_*i*_, ($$i={b}_{0^{\prime} }{b}_{0^{\prime\prime} },{b}_{0^{\prime} }{b}_{1},{b}_{0^{\prime} }{b}_{2}$$, and $${b}_{0^{\prime} }{b}_{3}$$), which were appropriately normalized assuming the following relations $${P}_{0}^{\exp }={P}_{1}^{\exp }={P}_{2}^{\exp }={P}_{3}^{\exp }={P}_{{b}_{0}{b}_{0}}^{\,{{\mbox{m}}}\,}$$, $${P}_{4}^{\exp }$$$$={P}_{5}^{\exp }={P}_{{b}_{0}{b}_{1}}^{\,{{\mbox{m}}}\,}$$, $${P}_{6}^{\exp }={P}_{7}^{\exp }={P}_{{b}_{0}{b}_{2}}^{\,{{\mbox{m}}}\,}$$, and $${P}_{8}^{\exp }={P}_{9}^{\exp }={P}_{{b}_{0}{b}_{3}}^{\,{{\mbox{m}}}\,}$$. Note that the assumed relations are always satisfied for *P*_*l*_(***φ***) with an ideal quarter and the experimentally reconstructed quarter transition matrix is close to an ideal quarter. Then obtained $${P}_{l}^{\exp }({{{{{{{\boldsymbol{\varphi }}}}}}}})$$ are functions of unknown phases ***φ*** and used to calculate the derivatives in Eq. (4). The detailed relation between $${P}_{l}^{\exp }({{{{{{{\boldsymbol{\varphi }}}}}}}})$$ and $${P}_{{b}_{i}{b}_{j}}^{\,{{\mbox{m}}}\,}$$ are provided in Supplementary Note 3.

**Fig. 4:**
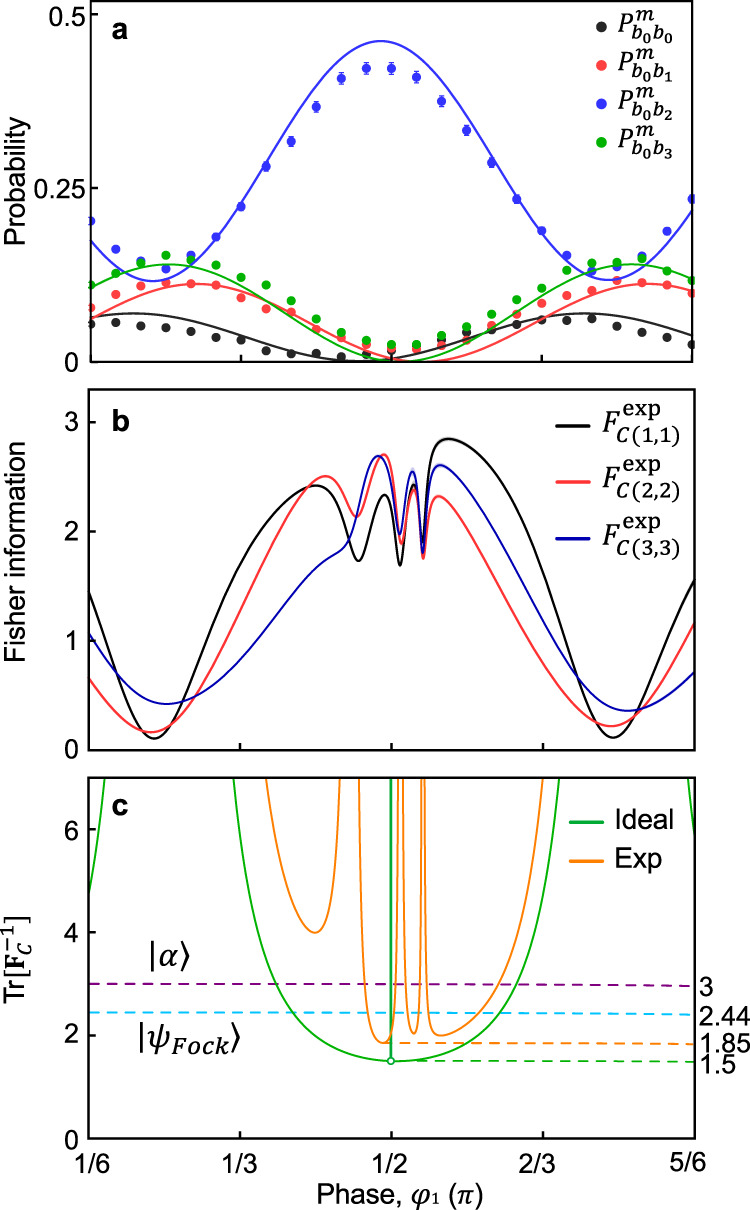
Measured two-photon detection probabilities with scanning *φ*_1_ and the corresponding Fisher information and CRB. **a** ${P}_{{b}_{0}{b}_{0}}^{\,{{\mbox{m}}}\,}$, ${P}_{{b}_{0}{b}_{1}}^{\,{{\mbox{m}}}\,}$, ${P}_{{b}_{0}{b}_{2}}^{\,{{\mbox{m}}}\,}$, and ${P}_{{b}_{0}{b}_{3}}^{\,{{\mbox{m}}}\,}$ (black, red, blue, and green, respectively) at *φ*_2_ = −0.07*π* and *φ*_3_ = 0.52*π*. Solid lines represent fitting functions using the ${\left|{\psi }_{4}^{2}\right\rangle }_{\exp }$ probe state and the experimentally reconstructed quarter transition matrix ${{{{{{{{\bf{U}}}}}}}}}_{q,\exp }$. **b** Corresponding orthogonal term of CFIM obtained from the measured probabilities ${P}_{{b}_{0}{b}_{0}}^{\,{{\mbox{m}}}\,}$, ${P}_{{b}_{0}{b}_{1}}^{\,{{\mbox{m}}}\,}$, ${P}_{{b}_{0}{b}_{2}}^{\,{{\mbox{m}}}\,}$, and ${P}_{{b}_{0}{b}_{3}}^{\,{{\mbox{m}}}\,}$ as a function of *φ*_1_. **c** Corresponding CRB values. Orange and green lines correspond to the experimental results and the theoretical calculation, respectively. Dashed purple and cyan lines refer to the minimum CRB values: 3 for a coherent state ($\overline{N}=2$) and 2.44 for $\left|{\psi }_{{{\mbox{Fock}}}}\right\rangle$ using an ideal quarter, respectively. All error bars represent one standard deviation and shaded areas correspond to the one standard deviation from uncertainty of the fitting parameter.

Experimentally obtained two-photon detection probabilities are shown in Fig. 4a where our experimental data are well-matched to our fitting function ${P}_{l}^{\exp }({{{{{{{\boldsymbol{\varphi }}}}}}}})$, which are obtained from ${\left|{\psi }_{4}^{2}\right\rangle }_{\exp }$ and ${{{{{{{{\bf{U}}}}}}}}}_{q,\exp }$ (see “Methods”). The CFIM can be obtained by using ${P}_{l}^{\exp }({{{{{{{\boldsymbol{\varphi }}}}}}}})$, and the diagonal terms of the CFIM at various *φ*_1_ are plotted in Fig. 4b (see “Methods” for the detailed information). Note that the maximum values of all diagonal terms of the CFIM are 3 at *φ*_1_ ≃ 0.5*π* with an ideal $\left|{\psi }_{4}^{2}\right\rangle$ state and an ideal quarter. Then we numerically find the minimum CRB of $\,{{\mbox{Tr}}}\,[{({{{{{{{{\bf{F}}}}}}}}}_{C}^{\exp })}^{-1}({{{{{{{\boldsymbol{\varphi }}}}}}}})]=1.85\pm 0.01$ when *φ*_1_ ≃ 0.47*π*, *φ*_2_ = −0.07*π*, and *φ*_3_ = 0.52*π* from ${P}_{l}^{\exp }({{{{{{{\boldsymbol{\varphi }}}}}}}})$, which is clearly smaller than the CRB of the coherent state $\,{{\mbox{Tr}}}\,[{({{{{{{{{\bf{F}}}}}}}}}_{C,{{{{{{{\rm{coh}}}}}}}}})}^{-1}({{{{{{{\boldsymbol{\varphi }}}}}}}})]=3$ with the same average photon number $\overline{N}=2$ as shown in Fig. 4c (see Supplementary Note 3 for the detailed calculation on estimating the CRB and results with respect to *φ*_2_ and *φ*_3_.). Note that the ideal CRB value can achieve 1.5 to saturate the QCRB with an ideal $\left|{\psi }_{4}^{2}\right\rangle$ state and an ideal quarter at *φ*_1_ ≃ 0.5*π*, *φ*_2_ = 0, and *φ*_3_ = 0.5*π*.

In our experiment, experimental errors are mainly from non-unity visibility of the observed interference, a phase fluctuation in each arm of an interferometer, a normalization assumption due to lack of superconducting nanowire single-photon detector (SNSPD) channels, and the fact that the quarter (${{{{{{{{\bf{U}}}}}}}}}_{q,\exp }$) used in our experiment is slightly different from an ideal quarter **U**_*q*_, see Methods for comparison between ${{{{{{{{\bf{U}}}}}}}}}_{q,\exp }$ and **U**_*q*_. In particular, in Eq. (4), with an ideal quarter, ∂*P*_*l*_(***φ***)/∂*φ*_*j*_ = 0 should be at the same *φ*_*j*_ value for all *P*_*l*_(***φ***), however, a non-ideal quarter transition matrix ${{{{{{{{\bf{U}}}}}}}}}_{q,\exp }$ makes $\partial {P}_{l}^{\exp }({{{{{{{\boldsymbol{\varphi }}}}}}}})/\partial {\varphi }_{j}=0$ happens at slightly different *φ*_*j*_ values. One can notice it from Fig. 4a that ${P}_{{b}_{0}{b}_{0}}^{\,{{\mbox{m}}}\,}$, ${P}_{{b}_{0}{b}_{1}}^{\,{{\mbox{m}}}\,}$, and ${P}_{{b}_{0}{b}_{3}}^{\,{{\mbox{m}}}\,}$ (${P}_{{b}_{0}{b}_{2}}^{\,{{\mbox{m}}}\,}$) do not have their minimum (maximum) value at *φ*_1_ = 0.5*π*. This is the reason why the minimum value of the CRB is not obtained at *φ*_1_ ≃ 0.5*π* but *φ*_1_ ≃ 0.47*π*.

Furthermore, we emphasize that the enhanced sensitivity can be obtained by the prepared $\left|{\psi }_{4}^{2}\right\rangle$ probe state compared to other quantum probe states using single-photon Fock states $\left|{\psi }_{{{\mbox{Fock}}}}\right\rangle$ proposed in ref. ^24^, where $\left|1100\right\rangle$ state is used as an input state instead of $\left|\alpha \right\rangle$ in Fig. 1a (detailed calculations are provided in Supplementary Note 2). Moreover, we theoretically compare the sensitivity bounds between our multi-mode *N*00*N* state and an amplitude-unbalanced multi-mode *N*00*N* state $\left|{\psi }_{u}\right\rangle$, which is proposed in refs. ^14,15,39^. The amplitude-unbalanced multi-mode *N*00*N* state has the form of $\left|{\psi }_{u}\right\rangle =\alpha \left|N0\cdots 0\right\rangle +\beta (\left|0N0\cdots 0\right\rangle +\cdots +\left|0\cdots 0N\right\rangle )$ with *α*^2^ + *dβ*^2^ = 1 and $\beta =1/\sqrt{d+\sqrt{d}}$. $\left|{\psi }_{u}\right\rangle$ is known to have the minimum QCRB among the multi-mode *N*00*N* states^14,15,39^. In a measurement scheme using a quarter and PNRDs, the QCRB and the CRB values of $\left|{\psi }_{u}\right\rangle$ with *N* = 2 and *d* = 3 are theoretically calculated to be 1.4 and 1.62, respectively. Here, we find that even though $\left|{\psi }_{u}\right\rangle$ has the lower QCRB of 1.4 than 1.5 of our $\left|{\psi }_{4}^{2}\right\rangle$, $\left|{\psi }_{4}^{2}\right\rangle$ can provide a better sensitivity (smaller CRB) 1.5 than 1.62 of $\left|{\psi }_{u}\right\rangle$ with a realistic measurement scheme using a quarter. Note that an optimal measurement saturating the QCRB may not be experimentally feasible^14,17^.

In Table 1, we summarize the ideal QCRB and CRB values for various probe states as well as the CRB values obtained from our experimental results. We emphasize that the experimentally obtained CRB value of 1.85 ± 0.01 provides a better sensitivity than the ideal CRB values of 3 for $\left|\alpha \right\rangle$ and 2.44 for $\left|{\psi }_{{{\mbox{Fock}}}}\right\rangle$.

**Table 1:** CRB and QCRB for total variances of ∑∣Δ*φ*∣^2^ with various probe states.

Probe state	*U*	QCRB	CRB
$\left|\alpha \right\rangle$ ($\overline{N}=2$)	**U**_*q*_	3	3
$\left|{\psi }_{{{\mbox{Fock}}}}\right\rangle$^24^	**U**_*q*_	2.33	2.44
$\left|{\psi }_{u}\right\rangle$^14^	**U**_*q*_	1.4	1.62
$\left|{\psi }_{4}^{2}\right\rangle$	**U**_*q*_	1.5	1.5
${\left|{\psi }_{4}^{2}\right\rangle }_{\exp }$	${{{{{{{{\bf{U}}}}}}}}}_{q,\exp }$	1.54 ± 0.01	1.85 ± 0.01

Our experiments use the post-selection technique and does not consider the photonic losses due to experimental imperfection^43^ and lack of high-efficiency PNRDs^36^. By considering the post-selection probability and photon losses, the CRB of our $\left|{\psi }_{4}^{2}\right\rangle$ state cannot outperform the classical strategy. However, a genuine quantum enhancement can be achieved if one uses the state-of-art technologies such as high-efficiency PNRDs and optimized low-loss optical components with our $\left|{\psi }_{4}^{2}\right\rangle$ state. Note that the post-selection technique does not affect the proof-of-concept verification of quantum enhancement and post-selection is standard technique used in almost previous quantum metrology experiment^15,23,24,29^. The corresponding quantitative analysis as well as the detailed discussions on photonic losses in our experimental setup are provided in Supplementary Note 4.

## Discussion

In conclusion, we proposed a scheme for generating a multi-mode *N*00*N* state and experimentally demonstrate that the prepared quantum probe state is the 4-mode 2002 state by observing various interference fringes shown in Fig. 3 using a quarter and photon number resolving detection using post-selective pseudo-PNRDs. Moreover, we exploit the prepared 4-mode 2002 state as a quantum probe state for simultaneously estimating three phases of a 4-mode interferometer with quantum enhanced sensitivity. Then, we confirm that the CRB obtained by our 4-mode 2002 state and measurement scheme can saturate the QCRB. Our results provide a practical platform to investigate intriguing issues in the field of quantum multiple parameter metrology. At first, we emphasize that our scheme can be extended to generation of higher-mode *N*00*N* states. For instance, one can exploit a multiple-path Sagnac interferometer^44^ to increase the number of modes from 4 to 4*n*, i.e., $\left|{\psi }_{4n}^{2}\right\rangle$, where *n* is the number of Sagnac interferometers, and then one can estimate up to 4*n* − 1 phases simultaneously. Note that another scheme for generating multi-mode *N*00*N* states with *N* ≥ 2 has been theoretically proposed^33^. However, experimental demonstration seems to be challenging within current technology since it requires extremely strong nonlinearity or deterministic generation of multi-photon states. Another interesting future direction would be finding a realistic measurement scheme for minimizing the CRB. In general, the CRB obtained by measurement scheme using a balanced multi-mode beam splitter cannot saturate the QCRB, see Fig. 1c with *d* = 2, 4, 5. Since the optimal measurement saturating the QCRB involves complex multi-photon states, it may not be experimentally feasible^14,17^. Hence, finding an experimentally realistic measurement scheme minimizing the CRB is essential for practical applications. Our results have direct applications for the quantum enhanced phase object imaging requiring a low photon flux^7,45,46^. In addition, our results can pave the way for demonstrating distributed quantum enhanced multiple-phase estimation by increasing the number of phases in local interferometers consisting of distributed quantum sensors^12,23,27^.

## Methods

### 4-mode 2002 state preparation

We used a CW single frequency laser operating at a center wavelength of 780 nm. The polarization of pump laser is set to $\left|D\right\rangle =\left(\left|H\right\rangle +\left|V\right\rangle \right)/\sqrt{2}$ polarization. The 10 mm-thick type-II periodically poled KTiOPO_4_ (PPKTP) crystal with 46.15 μm poling period is located at the center of the Sagnac interferometer, which consists of a dual wavelength polarizing beam splitter (PBS), a dual wavelength HWP whose optic axis is oriented at 45^∘^, and two dual wavelength mirrors as shown in Fig. 2c^47^. Here, dual wavelength optical components are designed for working at both 780 and 1560 nm photons. The horizontal (vertical) polarization component of the pump laser is transmitted (reflected) at dual wavelength PBS. The vertically polarized pump laser is changed to the horizontal polarization after transmitting a dual wavelength HWP. Then, the both of clockwise and counter-clockwise propagating pump laser have horizontal polarization in front of the PPKTP crystal and they probabilistically create photon pairs having orthogonal polarizations of $\left|H\right\rangle$ and $\left|V\right\rangle$, respectively, via degenerate spontaneous parametric down conversion process. Then, after passing through the dual wavelength PBS, the counter propagating photon pair states are emerging to $(\left|HV\right\rangle +{e}^{i\phi }\left|VH\right\rangle )/\sqrt{2}$, where *ϕ* is relative phase between two states. Then, we can prepare $\left|{{{\Phi }}}^{+}\right\rangle$ state using a set of WPs. The measured heralding efficiency of $\left|{{{\Phi }}}^{+}\right\rangle$ state is 37% with our SNSPD whose detection efficiency is 80%.

Two photons prepared in $\left|{{{\Phi }}}^{+}\right\rangle$ state simultaneously entered at both input ports of a lateral beam splitter (LBS), then we can observe the Hong-Ou-Mandel (HOM) interference^48^. The path length difference between two photons are matched to minimize the coincidence count rate detected at both output ports. See Supplementary Note 1 for the HOM interference results with $\left|{{{\Phi }}}^{+}\right\rangle$ input state. After the LBS, we can prepare the two-photon *N*00*N* state with different polarization (See the second line of Eq. (3)). The horizontally (vertically) polarized photons are transmitted (reflected) at the PBS so that they split into 4 path modes depending on the polarization states. Note that only two-photon states can exist in each mode. By rotating the polarization state of the vertically polarized photons into the horizontal polarization, we can prepare $\left|{\psi }_{4}^{2}\right\rangle$.

### Theoretical analysis of $\left|{\psi }_{4}^{2}\right\rangle$

Our probe state is the 4-mode 2002 state of the form

5 $$\left|{\psi }_{4}^{2}\right\rangle =\frac{1}{2\sqrt{2}}({\hat{a}}_{{{{{{{{\rm{0}}}}}}}}}^{{{{\dagger}}} }{\hat{a}}_{{{{{{{{\rm{0}}}}}}}}}^{{{{\dagger}}} }+{\hat{a}}_{{{{{{{{\rm{1}}}}}}}}}^{{{{\dagger}}} }{\hat{a}}_{{{{{{{{\rm{1}}}}}}}}}^{{{{\dagger}}} }+{\hat{a}}_{{{{{{{{\rm{2}}}}}}}}}^{{{{\dagger}}} }{\hat{a}}_{{{{{{{{\rm{2}}}}}}}}}^{{{{\dagger}}} }+{\hat{a}}_{{{{{{{{\rm{3}}}}}}}}}^{{{{\dagger}}} }{\hat{a}}_{{{{{{{{\rm{3}}}}}}}}}^{{{{\dagger}}} })\left|0\right\rangle ,$$

where ${\hat{a}}_{{{{{{{{\rm{0}}}}}}}}}^{{{{\dagger}}} }$ is a creation operator, which creates a single-photon in the input mode *a*_0_ of a quarter. A quarter has four input modes (*a*_0_, *a*_1_, *a*_2_, *a*_3_) and four output modes (*b*_0_, *b*_1_, *b*_2_, *b*_3_), respectively, as shown in Fig. 2c. The unitary matrices for the phase encoding **U**_*φ*_ and the ideal quarter transition **U**_*q*_ are given by^37,38^,

6 $${{{{{{{{\bf{U}}}}}}}}}_{\varphi }=\left(\begin{array}{cccc}1&0&0&0\\ 0&{e}^{i{\varphi }_{1}}&0&0\\ 0&0&{e}^{i{\varphi }_{2}}&0\\ 0&0&0&{e}^{i{\varphi }_{3}}\end{array}\right),$$

and

7 $${{{{{{{{\bf{U}}}}}}}}}_{q}=\frac{1}{2}\left(\begin{array}{cccc}1&1&1&1\\ 1&1&-1&-1\\ 1&-1&1&-1\\ 1&-1&-1&1\end{array}\right),$$

respectively. After the initial state $\left|{\psi }_{4}^{2}\right\rangle$ undergoes the phase encoding and the quarter transformation, then the output state $\left|{\psi }_{{{\mbox{out}}}}\right\rangle ={{{{{{{{\bf{U}}}}}}}}}_{q}{{{{{{{{\bf{U}}}}}}}}}_{\varphi }\left|{\psi }_{4}^{2}\right\rangle$ becomes

8 $$\begin{array}{ll}&\left|{\psi }_{{{\mbox{out}}}}\right\rangle ={c}_{0}\left|2000\right\rangle +{c}_{1}\left|0200\right\rangle +{c}_{2}\left|0020\right\rangle +{c}_{3}\left|0002\right\rangle \\ &+\,{c}_{4}\left|1100\right\rangle +{c}_{5}\left|0011\right\rangle +{c}_{6}\left|1010\right\rangle \\ &+\,{c}_{7}\left|0101\right\rangle +{c}_{8}\left|1001\right\rangle +{c}_{9}\left|0110\right\rangle ,\end{array}$$

with *c*_0_ = *c*_1_ = *c*_2_ = *c*_3_, *c*_4_ = *c*_5_, *c*_6_ = *c*_7_, and *c*_8_ = *c*_9_. Then, one can obtain the two-photon detection probability of *P*_*l*_(***φ***) = ∣*c*_*l*_∣^2^ (*l* = 0, 1, ..., 9), and it satisfies the normalizing condition ∑_*l*_*P*_*l*_(***φ***) = 1. *P*_*l*_(***φ***) is a function of ***φ***, and used for theoretical calculations in Fig. 3. See Supplementary Note 3 for the detailed information on *P*_*l*_(***φ***) = ∣*c*_*l*_∣^2^.

### Analysis considering experimental errors

In order to include the errors caused by our experimental imperfection, we consider the generation of 4-mode 2002 state ${\left|{\psi }_{4}^{2}\right\rangle }_{\exp }$ from an imperfect Bell state of $|{{{\Phi }}}_{\exp }^{+}\rangle$, which is given by^49^

9 $$|{{{\Phi }}}_{\exp }^{+}\rangle =\; \epsilon \frac{1}{\sqrt{2}}({\hat{\alpha }}_{0}^{H{{{\dagger}}} }{\hat{\alpha }_{1}^{H{{{\dagger}}} }}+{\hat{\alpha }}_{0}^{V{{{\dagger}}} }{\hat{\alpha }_{1}^{V{{{\dagger}}} }})\left|0\right\rangle \\ +\,\sqrt{1-| \epsilon {| }^{2}}\frac{1}{\sqrt{2}}({\hat{\alpha }}_{0}^{^{\prime} H{{{\dagger}}} }{\hat{\alpha }_{1}^{H{{{\dagger}}} }}+{\hat{\alpha }}_{0}^{^{\prime} V{{{\dagger}}} }{\hat{\alpha }_{1}^{V{{{\dagger}}}} })\left|0\right\rangle,$$

where ${\hat{\alpha }}_{j}^{H{{{\dagger}}} }$ (${\hat{\alpha }}_{j}^{V{{{\dagger}}} }$) creates a photon at *α*_*j*_ with a horizontal (vertical) polarization shown in Fig. 2c, and ${\hat{\alpha }}_{j}^{^{\prime} H{{{\dagger}}} }$ (${\hat{\alpha }}_{j}^{^{\prime} V{{{\dagger}}} }$) creates a distinguishable photon with ${\hat{\alpha }}_{j}^{H{{{\dagger}}} }$ (${\hat{\alpha }}_{j}^{V{{{\dagger}}} }$) at same position due to experimental imperfection. Note that photons created from ${\hat{\alpha }}_{j}^{H{{{\dagger}}} }$ (${\hat{\alpha }}_{j}^{V{{{\dagger}}} }$) and ${\hat{\alpha }}_{j}^{^{\prime} H{{{\dagger}}} }$ (${\hat{\alpha }}_{j}^{^{\prime} V{{{\dagger}}} }$) are distinguishable and they do not interfere each other, and *ϵ* is real parameter varying from 0 to 1. Then after LBS, PBS, and HWP at 45^∘^, the experimentally prepared ${\left|{\psi }_{4}^{2}\right\rangle }_{\exp }$ becomes

10 $${\left|{\psi }_{4}^{2}\right\rangle }_{\exp }=\; \epsilon \frac{1}{2\sqrt{2}}({\hat{a}}_{0}^{{{{\dagger}}} }{\hat{a}}_{0}^{{{{\dagger}}} }+{\hat{a}}_{1}^{{{{\dagger}}} }{\hat{a}}_{1}^{{{{\dagger}}} }+{\hat{a}}_{2}^{{{{\dagger}}} }{\hat{a}}_{2}^{{{{\dagger}}} }+{\hat{a}}_{3}^{{{{\dagger}}} }{\hat{a}}_{3}^{{{{\dagger}}} })\left|0\right\rangle \\ +\,\sqrt{1-| \epsilon {| }^{2}}\frac{1}{2\sqrt{2}}\left({\hat{a}}_{0}^{{{{\dagger}}} }{\hat{a}}_{0}^{^{\prime} {{{\dagger}}} }+{\hat{a}}_{1}^{{{{\dagger}}} }{\hat{a}}_{1}^{^{\prime} {{{\dagger}}} }+{\hat{a}}_{2}^{{{{\dagger}}} }{\hat{a}}_{2}^{^{\prime} {{{\dagger}}} }+{\hat{a}}_{3}^{{{{\dagger}}} }{\hat{a}}_{3}^{^{\prime} {{{\dagger}}} }\right.\\ +\,i\left.\left({\hat{a}}_{0}^{^{\prime} {{{\dagger}}} }{\hat{a}}_{1}^{{{{\dagger}}} }+{\hat{a}}_{0}^{{{{\dagger}}} }{\hat{a}}_{1}^{^{\prime} {{{\dagger}}} }+{\hat{a}}_{2}^{^{\prime} {{{\dagger}}} }{\hat{a}}_{3}^{{{{\dagger}}} }+{\hat{a}}_{2}^{{{{\dagger}}} }{\hat{a}}_{3}^{^{\prime} {{{\dagger}}} }\right)\right)\left|0\right\rangle ,$$

where ${\hat{a}}_{i}^{{{{\dagger}}} }$ creates a photon at input port of quarter *a*_*i*_ and ${\hat{a}}_{i}^{^{\prime} {{{\dagger}}} }$ creates a distinguishable photon due to experimental imperfection. Note that photons created from ${\hat{a}}_{i}^{{{{\dagger}}} }$ and ${\hat{a}}_{i}^{^{\prime} {{{\dagger}}} }$ are distinguishable and they do not interfere each other. After ${\left|{\psi }_{4}^{2}\right\rangle }_{\exp }$ undergoes the phase encoding **U**_*φ*_ and the experimentally reconstructed quarter transition matrix ${{{{{{{{\bf{U}}}}}}}}}_{q,\exp }$, then the output state becomes ${\left|{\psi }_{{{\mbox{out}}}}\right\rangle }_{\exp }={{{{{{{{\bf{U}}}}}}}}}_{q,\exp }{{{{{{{{\bf{U}}}}}}}}}_{\varphi }{\left|{\psi }_{4}^{2}\right\rangle }_{\exp }$. Here, ${{{{{{{{\bf{U}}}}}}}}}_{q,\exp }$ is given by

11 $${{{{{{{{\bf{U}}}}}}}}}_{q,\exp }=\left(\begin{array}{cccc}0.498&0.469&0.514&0.478\\ 0.483&0.496&0.504{e}^{i0.261}&-0.509\\ 0.529&0.504{e}^{i0.244}&0.505{e}^{i0.126}&0.499{e}^{i0.346}\\ 0.489&-0.530&0.477{e}^{i0.356}&0.516\end{array}\right).$$

Then experimental two-photon detection probabilities are obtained as ${P}_{l}^{\exp }({{{{{{{\boldsymbol{\varphi }}}}}}}})$, it is used for fitting curves in Fig. 4a. See Supplementary Note 3 for the detailed calculations on our error analysis.

### Quantum Fisher information matrix and CRB

The QFIM is given by

12 $${F}_{Q(j,k)}=4\,{{\mbox{Re}}}\,(\langle {\partial }_{{\varphi }_{j}}{\psi }_{\varphi }| {\partial }_{{\varphi }_{k}}{\psi }_{\varphi }\rangle -\langle {\partial }_{{\varphi }_{j}}{\psi }_{\varphi }| {\psi }_{\varphi }\rangle \langle {\psi }_{\varphi }| {\partial }_{{\varphi }_{k}}{\psi }_{\varphi }\rangle ).$$

By calculating **F**_*Q*_, one can obtain the QCRB from $\,{{\mbox{Tr}}}\,[{{{{{{{{\bf{F}}}}}}}}}_{Q}^{-1}]$^14,16,17,39^. For a $\left|{\psi }_{4}^{2}\right\rangle$ probe state, the QCRB is calculated to be $\,{{\mbox{Tr}}}\,[{{{{{{{{\bf{F}}}}}}}}}_{Q}^{-1}]$ = 1.5, and the minimum value of the CRB is obtained at *φ*_1_ ≃ 0.5*π*, *φ*_2_ = 0, and *φ*_3_ = 0.5*π*, and it saturates the QCRB with $\,{{\mbox{Tr}}}\,[{{{{{{{{\bf{F}}}}}}}}}_{Q}^{-1}]$ = $\,{{\mbox{Tr}}}\,[{{{{{{{{\bf{F}}}}}}}}}_{C}^{-1}({{{{{{{\boldsymbol{\varphi }}}}}}}})]$ = 1.5. The ideal **F**_*C*_(***φ***) and **F**_*Q*_ matrices are obtained to be

13 $${{{{{{{{\bf{F}}}}}}}}}_{C}({{{{{{{\boldsymbol{\varphi }}}}}}}})={{{{{{{{\bf{F}}}}}}}}}_{Q}=\left(\begin{array}{lll}3&-1&-1\\ -1&3&-1\\ -1&-1&3\end{array}\right).$$

where *φ*_1_ ≃ 0.5*π*, *φ*_2_ = 0, and *φ*_3_ = 0.5*π*.

The experimentally obtained ${{{{{{{{\bf{F}}}}}}}}}_{C}^{\exp }({{{{{{{\boldsymbol{\varphi }}}}}}}})$ matrix using ${P}_{l}^{\exp }({{{{{{{\boldsymbol{\varphi }}}}}}}})$ is given as below:

14 $${{{{{{{{\bf{F}}}}}}}}}_{C}^{\exp }({{{{{{{\boldsymbol{\varphi }}}}}}}})=\left(\begin{array}{lll}2.33&-0.63&-0.93\\ -0.63&2.70&-1.09\\ -0.93&-1.09&2.66\end{array}\right).$$

where *φ*_1_ ≃ 0.47*π*, *φ*_2_ = −0.07*π*, and *φ*_3_ = 0.52*π*. The experimentally obtained CRB is evaluated to be 1.85 ± 0.01. The detailed calculations are provided in Supplementary Note 3.

## Supplementary information

Supplementary Information

## Data Availability

The data that support the findings of this study are available from the corresponding author upon reasonable request.

## Code availability

The code used to generate the figures within this paper and other findings of this study are available from the corresponding author upon reasonable request.
