# Author Correction: Quantum enhanced multiple-phase estimation with multi-mode *N*00*N* states

**DOI:** 10.1038/s41467-021-27483-2

**Published:** 2021-12-06

**Authors:** Seongjin Hong, Junaid ur Rehman, Yong-Su Kim, Young-Wook Cho, Seung-Woo Lee, Hojoong Jung, Sung Moon, Sang-Wook Han, Hyang-Tag Lim

**Affiliations:** 1 Center for Quantum Information, Korea Institute of Science and Technology (KIST), Seoul, Korea; 2 Department of Electronics and Information Convergence Engineering, Kyung Hee University, Yongin, Korea; 3 Division of Nano and Information Technology, KIST School, Korea University of Science and Technology, Seoul, Korea; 4 Department of Physics, Yonsei University, Seoul, Korea

**Keywords:** Quantum optics, Quantum information, Quantum metrology

Correction to: *Nature Communications* 10.1038/s41467-021-25451-4, published online 01 September 2021.

The original version of this Article contained an error in the author affiliations. The affiliation of Young-Wook Cho with Department of Physics, Yonsei University, Seoul, Korea was inadvertently omitted. The correct affiliations should read: Center for Quantum Information, Korea Institute of Science and Technology (KIST), Seoul, Korea; Department of Physics, Yonsei University, Seoul, Korea

This has now been corrected in both the PDF and HTML versions of the Article.

