# Supplementary material for: Quantum enhanced multiple-phase estimation with multi-mode N00N states
Source: Nat Commun. 2021 Sep 1;12:5211. doi: 10.1038/s41467-021-25451-4 (PMC8410777; doi:10.1038/s41467-021-25451-4)
Supplement: Supplementary file 1 — Supplementary Information [file 41467_2021_25451_MOESM1_ESM.pdf]

Supplementary Information - Quantum enhanced multiple phase estimation with multi-mode $N00N$ states

Seongjin Hong,[1] Junaid ur Rehman,[1,2] Yong-Su Kim,[1,3] Young-Wook Cho,[1] Seung-Woo Lee,[1] Hojoong Jung,[1] Sung Moon,[1,3] Sang-Wook Han,[1,3] and Hyang-Tag Lim[1,3,*]

[1]*Center for Quantum Information, Korea Institute of Science and Technology (KIST), Seoul, 02792, Republic of Korea*
[2]*Department of Electronics and Information Convergence Engineering, Kyung Hee University, Yongin, 17104, Korea*
[3]*Division of Nano and Information Technology, KIST School, Korea University of Science and Technology, Seoul 02792, Republic of Korea*

[*] hyangtag.lim@kist.re.kr

SUPPLEMENTARY NOTE 1 - EXPERIMENTAL DETAILS

A. Source preparation

We prepare the 4-mode 2002 state using the Hong-Ou-Mandel (HOM) interference from $|\Phi^+\rangle = (|HH\rangle + |VV\rangle)/\sqrt{2}$ triplet Bell state with fidelity $F = 0.977$. The measured HOM interference fringes for $|HH\rangle$ and $|VV\rangle$ of input states $|\Phi^+\rangle$ are shown in Supplementary Figure 1. The near unity HOM visibility for each polarization component guarantees that the photon pair moves together almost perfectly along either arm of the beam splitter.

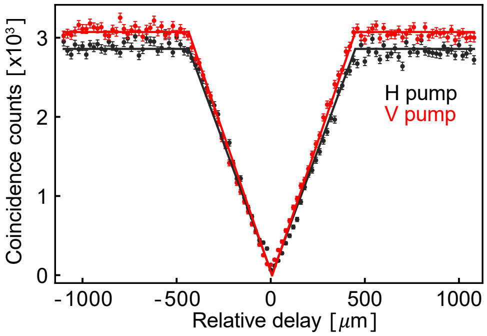

Supplementary Figure 1. **Experimental HOM interference.** The measured HOM visibilities are 0.993 and 0.998 for $|HH\rangle$ and $|VV\rangle$ input states, respectively. All error bars represent one standard deviation.

Then, we can prepare the 4-mode 2002 state after polarizing beam splitter (PBS) and half waveplate (HWP) as shown in Figure 2**a** of the main text. We measure the two-photon coincidence counts when we open two input modes and block two other modes of the generated the 4-mode 2002 state. We observed two times faster interference modulations between a_0&a_1, a_0&a_2, a_0&a_3, a_1&a_2, a_1&a_3, and a_2&a_3 as shown in Supplementary Figure 2.

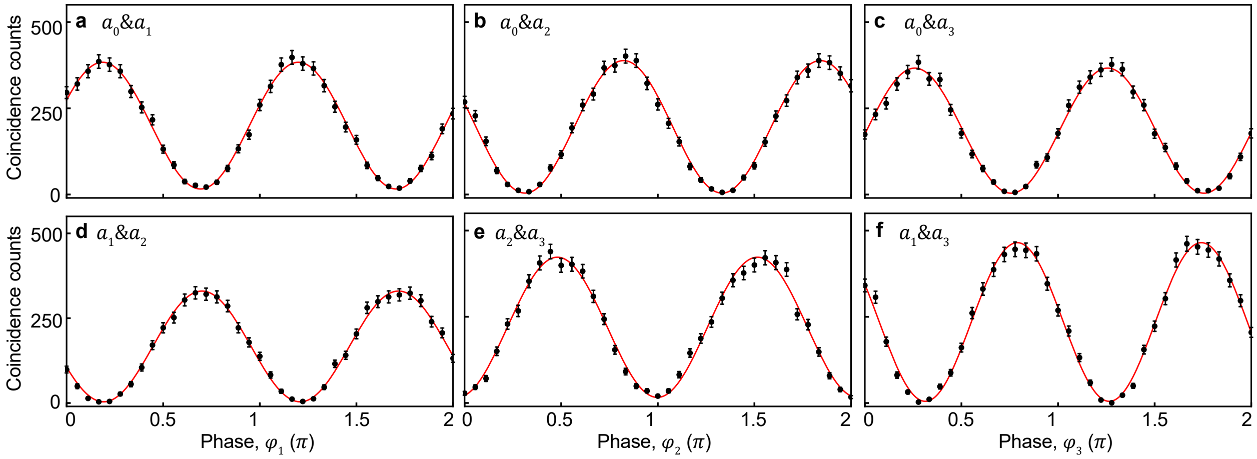

Supplementary Figure 2. **Interference fringes for the 4-mode 2002 state.** Two photon coincidence counts of two input modes among four modes of the input state with a visibility of **a,** 0.920 for a_0&a_1, **b,** 0.980 for a_0&a_2, **c,** 0.979 for a_0&a_3, **d,** 0.978 for a_1&a_2, **e,** 0.926 for a_2&a_3, and **f,** 0.979 for a_1&a_3, respectively. All error bars represent one standard deviation.

B. Reconstruction of the transition matrix of the quarter

To characterize the transition matrix of the experimentally implemented 4×4 multi-mode beam splitter, which is called a quarter, we implemented the reconstruction method proposed in Ref [3, 4]. The transition matrix of the ideal

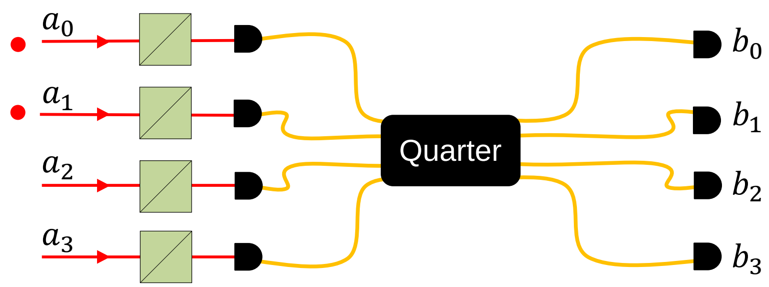

Supplementary Figure 3. **Experimental setup for two photon quantum interference measurements in a quarter.** All possible output coincidence ($C_{b_0b_1}$, $C_{b_0b_2}$, $C_{b_0b_3}$, $C_{b_1b_2}$, $C_{b_1b_3}$, and $C_{b_2b_3}$) are measured for each input state ($|1100\rangle$, $|1010\rangle$, $|1001\rangle$, $|0110\rangle$, $|0101\rangle$, and $|0011\rangle$).

quarter is given by

$$\mathbf{U}_q(\theta) = \frac{1}{2}\begin{pmatrix} 1 & 1 & 1 & 1 \\ 1 & e^{i\theta} & -1 & -e^{i\theta} \\ 1 & -1 & 1 & -1 \\ 1 & -e^{i\theta} & -1 & e^{i\theta} \end{pmatrix}, \tag{1}$$

where θ is a free internal phase. Let us define the transition matrix of a quarter, $\mathbf{U}$, as the following,

$$\mathbf{U} = \begin{pmatrix} |U_{0,0}| & |U_{0,1}| & |U_{0,2}| & |U_{0,3}| \\ |U_{1,0}| & |U_{1,1}|e^{i\theta} & |U_{1,2}|e^{i\phi_0} & -|U_{1,3}|e^{i\theta} \\ |U_{2,0}| & |U_{2,1}|e^{i\phi_1} & |U_{2,2}|e^{i\phi_2} & |U_{2,3}|e^{i\phi_3} \\ |U_{3,0}| & -|U_{3,1}|e^{i\theta} & |U_{3,2}|e^{i\phi_4} & |U_{3,3}|e^{i\theta} \end{pmatrix}, \tag{2}$$

whose elements U_{ij} is the transition amplitude of a photon entering in input port i and exiting at output port j. Note that our experimental quarter has the internal phase of $\theta \sim 0$. Then, we characterize the quarter operation by injecting two single-photons in two different input modes of a quarter such as either one of $|1100\rangle$, $|1010\rangle$, $|1001\rangle$, $|0110\rangle$, $|0101\rangle$, and $|0011\rangle$ as shown in Supplementary Figure 3. In the classical case that two distinguishable photons are injected into input ports of i and j, the probability of detecting one photon in each output port k and l is given by

$$C_{ij}^{kl} = |U_{ik}U_{jl}|^2 + |U_{il}U_{jk}|^2, \tag{3}$$

and in the classical case, there is no interference between the two single-photons. In the quantum case of two indistinguishable photons, quantum interference is revealed and it gives either a peak or a dip in the output coincidence probabilities. The detection probability of one photon in each output port k and l when two indistinguishable photons are injected in input ports of i and j is given by

$$Q_{ij}^{kl} = \frac{1}{1+\delta_{ij}}|U_{ik}U_{jl} + U_{il}U_{jk}|^2, \tag{4}$$

where δ_{ij} is Kronecker's delta. Using detection probabilities of C_{ij}^{kl} and Q_{ij}^{kl}, the visibility V_{ij}^{kl} of a non-classical peak or dip becomes

$$V_{ij}^{kl} = \frac{C_{ij}^{kl} - Q_{ij}^{kl}}{C_{ij}^{kl}}. \tag{5}$$

The transition matrix of a quarter $\mathbf{U}$ can be fully reconstructed via measurements of the transition amplitude of $|U_{ij}|^2$ and two-photon HOM visibilities of V_{ij}^{kl} for all possible combinations.

At first, we measure the count rate of $|U_{ij}|^2$ by injecting a single photon in only one input port. When i is fixed, $|U_{ij}|^2$ are obtained with the normalization condition of $\sum_{j=0}^{3}|U_{ij}|^2 = 1$ and the measured $|U_{ij}|^2$ is given by

$$|U_{ij}|^2 = \begin{pmatrix} 0.497 & 0.484 & 0.528 & 0.489 \\ 0.470 & 0.496 & 0.503 & 0.529 \\ 0.513 & 0.503 & 0.506 & 0.478 \\ 0.479 & 0.508 & 0.498 & 0.515 \end{pmatrix}. \tag{6}$$

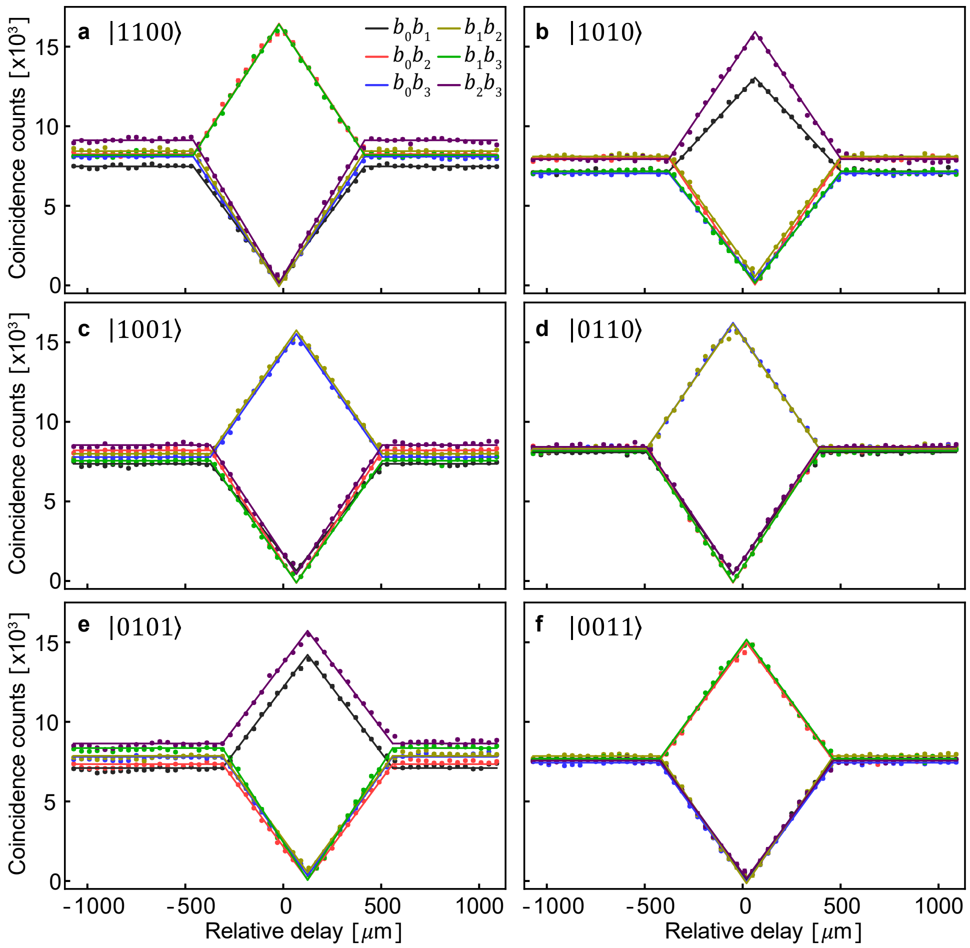

Supplementary Figure 4. **Experimental two-photon HOM interference results for various input states in a quarter.** **a,** $|1100\rangle$, **b,**$|1010\rangle$, **c,** $|1001\rangle$, **d,**$|0110\rangle$, **e,** $|0101\rangle$, and **f,** $|0011\rangle$ input states. All error bars represent one standard deviation.

Then, we measured the two-photon HOM visibility V_{ij}^{kl} corresponding to all possible combinations of the six input states and the six output two-photon coincidence counts as a function of the relative arrival time of the photons. Supplementary Figure 4 shows the HOM interference fringes for all combinations of the input states and the output ports, and we can obtain the experimentally measured HOM visibility V^m. On the other hand, by substituting the $|U_{ij}|$ of Supplementary Equation (2) with measured $|U_{ij}|^2$ of Supplementary Equation (6), we can obtain the analytic HOM visibility V^r. In order to reconstruct the transition matrix of quarter, we numerically minimize the distance between V^r and V^m by optimizing both phases ϕ_i and transition amplitude $|U_{ij}|$ with fixed internal phase $\theta = 0$. The reconstructed transition matrix of our quarter is calculated to be

$$\mathbf{U}_{q,\text{exp}} = \begin{pmatrix} 0.498 & 0.469 & 0.514 & 0.478 \\ 0.483 & 0.496 & 0.504e^{i0.261} & -0.509 \\ 0.529 & 0.504e^{i0.244} & 0.505e^{i0.126} & 0.499e^{i0.346} \\ 0.489 & -0.530 & 0.477e^{i0.356} & 0.516 \end{pmatrix}, \tag{7}$$

and graphically shown in Supplementary Figure 5.

To quantify the quality of the reconstructed transition matrix, we evaluate the similarity $S = 1 - \sum |V^r - V^m|/72$ [3, 4] between HOM visibilities from our experimental results and the reconstructed transition matrix. We obtained $S = 0.983$, which shows a good agreement with an ideal quarter matrix.

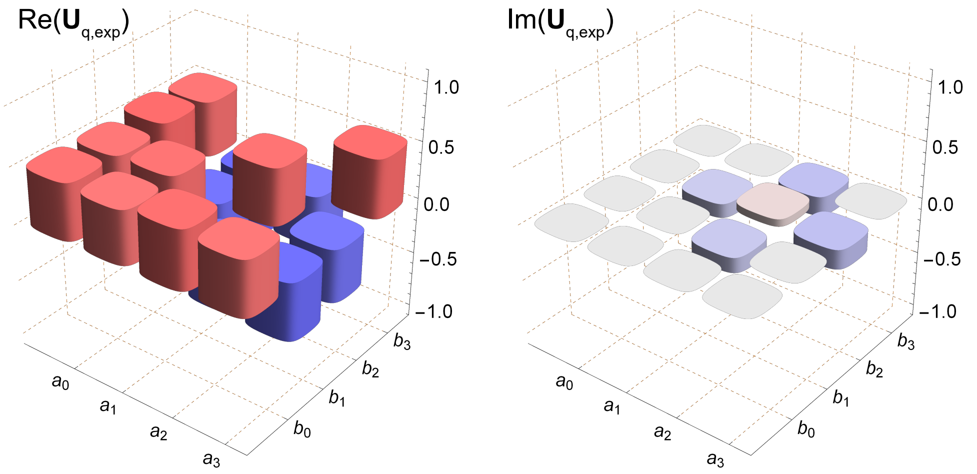

Supplementary Figure 5. **Reconstructed transition matrix of quarter. a,** Real and **b,** imaginary parts of the reconstructed matrix $\mathbf{U}_{q,\text{exp}}$.

SUPPLEMENTARY NOTE 2 - TOTAL VARIANCE OF DIFFERENT PROBE STATES WITH FIXED PHOTON NUMBER

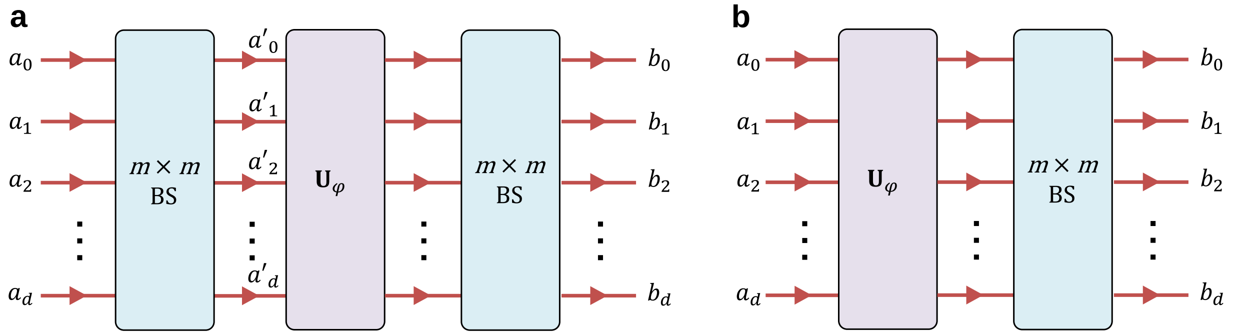

Supplementary Figure 6. **Multiple phase estimation schemes with different probe states. a,** Experimental scheme for coherent states and single-photon Fock states. **b,** Multiphase estimation scheme with multi-mode $N00N$ states.

We consider three types of probe states as shown in the main text: 1) single-photon Fock state $|\psi_{\text{Fock}}\rangle$ which can be prepared by injecting two-photon state $|110...0\rangle$ into the first and the second input port of an $m \times m$ multi-mode beam splitter, 2) a coherent state $|\alpha\rangle$ with an average photon number $\overline{N} = 2$, which is a classical probe state, and 3) an m-mode 2002 state $|\psi_m^2\rangle$ defined in Eq. (1) in main text.

A. Single-photon Fock states

At first, we consider single-photon Fock states $|\psi_{\text{Fock}}\rangle$. The initial state has the form

$$|\psi_{\text{in}}\rangle = \hat{a}_0^\dagger \hat{a}_1^\dagger |0\rangle, \tag{8}$$

where the operator $\hat{a}_i^\dagger$ creates a photon at i-th input port of an $m\times m$ multi-mode beam splitter, $\mathbf{U}_{\text{BS}}$ in Supplementary Figure 6**a**, and $|0\rangle$ is the vacuum. The matrix elements of $\mathbf{U}_{\text{BS}}$ and the phase encoding matrix $\mathbf{U}_\varphi$ are given by [1]

$$U_{\text{BS}}(j,k) = \frac{1}{\sqrt{m}} e^{2\pi ijk/m}, U_\varphi(j,k) = \delta_{jk} e^{i\varphi_j}, \tag{9}$$

where j and k vary between 0 and d ($m = d + 1$) and $\delta_{jk} = 1$ for $j = k$, otherwise $\delta_{jk} = 0$ and $\varphi_0 = 0$. $|\psi_{\text{Fock}}\rangle$ state can be prepared with the input state $|\psi_{\text{in}}\rangle$ after $\mathbf{U}_{\text{BS}}$ and it has the following form

$$|\psi_{\text{Fock}}\rangle = \mathbf{U}_{\text{BS}}|\psi_{\text{in}}\rangle = \sum_{j=0}^{d}\sum_{k=0}^{d} A_{jk}\hat{a'}_j^{\dagger}\hat{a'}_k^{\dagger}|0\rangle, \tag{10}$$

where A_{jk} is the probability amplitude of each output mode. Then the $|\psi_{\text{Fock}}\rangle$ undergoes phase shifts of $\mathbf{U}_{\varphi}$ and the phase encoded state $|\psi_{\varphi}\rangle$ becomes

$$|\psi_{\varphi}\rangle = \mathbf{U}_{\varphi}|\psi_{\text{Fock}}\rangle = \sum_{j=0}^{d}\sum_{k=0}^{d} A_{jk}(\boldsymbol{\varphi})\hat{a'}_j^{\dagger}\hat{a'}_k^{\dagger}|0\rangle, \tag{11}$$

where $A_{jk}(\boldsymbol{\varphi})$ has the information of the encoded phases $\boldsymbol{\varphi} = \{\varphi_1, \varphi_2, \cdots, \varphi_d\}$. We can obtain the quantum Fisher information matrix (QFIM) with $|\psi_{\varphi}\rangle$ using the following relation [2].

$$F_{Q(j,k)} = 4\text{Re}(\langle\partial_{\varphi_j}\psi_{\varphi}|\partial_{\varphi_k}\psi_{\varphi}\rangle - \langle\partial_{\varphi_j}\psi_{\varphi}|\psi_{\varphi}\rangle\langle\psi_{\varphi}|\partial_{\varphi_k}\psi_{\varphi}\rangle), \tag{12}$$

and then the quantum Cramer-Rao bound (QCRB) can be obtained from $\text{Tr}[\mathbf{F}_Q^{-1}]$ and the QCRB values with d from 1 to 5 are shown in Supplementary Table I.

We now consider a practical measurement scheme with another multi-mode beam splitter $\mathbf{U}_{\text{BS}}$ and photon number resolving detector (PNRD) to estimate the encoded phases of the probe states. Note that the QCRB value is not dependent on the detection scheme while the classical Cramer-Rao bound (CRB) value is dependent on a specific detection scheme. We can calculate the classical Fisher information matrix (CFIM) using the output state $|\psi_{\text{out}}\rangle$ after the $|\psi_{\varphi}\rangle$ state undergoes another $\mathbf{U}_{\text{BS}}$ operation. $|\psi_{\text{out}}\rangle$ is given by

$$|\psi_{\text{out}}\rangle = \mathbf{U}_{\text{BS}}|\psi_{\varphi}\rangle = \sum_{j=0}^{d}\sum_{k=0}^{d} B_{jk}(\boldsymbol{\varphi})\hat{b}_j^{\dagger}\hat{b}_k^{\dagger}|0\rangle, \tag{13}$$

where j and k vary from 0 and d and the detection probability of each output mode $|B_{jk}(\boldsymbol{\varphi})|^2 = P_l(\boldsymbol{\varphi})$ satisfies the normalization condition $\sum_l P_l(\boldsymbol{\varphi}) = 1$. Here, l varies from 0 to $m(m+1)/2 - 1$, which is the number of all possible output detection cases. The CFIM can be obtained with $P_l(\boldsymbol{\varphi})$ using the following relation [2],

$$F_{C(j,k)} = \sum_{l=0}^{m(m+1)/2-1} \frac{1}{P_l(\boldsymbol{\varphi})}\left(\frac{\partial P_l(\boldsymbol{\varphi})}{\partial\varphi_j}\right)\left(\frac{\partial P_l(\boldsymbol{\varphi})}{\partial\varphi_k}\right). \tag{14}$$

Then, we numerically minimize the $\text{Tr}[\mathbf{F}_C^{-1}(\boldsymbol{\varphi})]$ by changing the phase shift of $\boldsymbol{\varphi}$. The CRB can be evaluated from minimum $\text{Tr}[\mathbf{F}_C^{-1}(\boldsymbol{\varphi})]$, and they are also provided in Supplementary Table I with d from 1 to 5.

B. Coherent states

Then, we consider coherent states as a classical probe state as shown in Figure 1**a** in main text. The coherent state $|\alpha\rangle$ with $\overline{N} = |\alpha|^2 = 2$ can be considered as two distinguishable photons at input port a_0 in Supplementary Figure 6**a**. First, we calculate the QCRB and the CRB values using initial state $|\psi_{\text{coh}}\rangle = \hat{a}_0^{\dagger}|0\rangle$ with $\overline{N} = |\alpha|^2 = 1$, then the CRB and the QCRB values for $\overline{N} = |\alpha|^2 = 2$ are obtained by multiplying 2 by the values of $\overline{N} = |\alpha|^2 = 1$. The results of QCRB and CRB of $|\alpha\rangle$ with $\overline{N} = |\alpha|^2 = 2$ are shown in Supplementary Table I with varying d from 1 to 5.

C. Multi-mode $N00N$ states

We now consider the multi-mode $N00N$ state with the mode number m and the photon number $N = 2$ as shown in Supplementary Figure 6**b**, $|\psi_m^2\rangle$ having the following form:

$$\frac{1}{\sqrt{2m}}(\hat{a}_0^{\dagger}\hat{a}_0^{\dagger} + \hat{a}_1^{\dagger}\hat{a}_1^{\dagger} + ... + \hat{a}_d^{\dagger}\hat{a}_d^{\dagger})|0\rangle = |\psi_m^2\rangle. \tag{15}$$

	$\|\alpha\rangle$		$\|\psi_{\mathrm{Fock}}\rangle$		$\|\psi_m^2\rangle$	
d	QCRB	CRB	QCRB	CRB	QCRB	CRB
1	0.5	0.5	0.25	0.25	0.25	0.25
2	1.5	1.93	1	1.30	0.75	0.97
3	3	3	2.33	2.44	1.50	1.50
4	5	5.5	4.17	5.29	2.50	2.75
5	7.5	9.76	6.5	9.33	3.75	4.88

Supplementary Table I. CRB and QCRB for total variances of $\sum|\Delta\varphi|^2$ depending on the number of phases d with different quantum probe states with $N=2$.

After the $|\psi_m^2\rangle$ state undergoes phase encoding, it becomes $|\psi'_\varphi\rangle = \mathbf{U}_\varphi|\psi_m^2\rangle$ including phase information. Then we can obtain the QFIM and the QCRB of the case using the m-mode 2002 state as a probe state from Supplementary Equation (12), and the QCRB results of the m-mode $N00N$ states are provided in Supplementary Table I.

The CFIM is calculated for the output state after phase shifts and a multi-mode beam splitter as $|\psi'_{\mathrm{out}}\rangle = \mathbf{U}_{\mathrm{BS}}\mathbf{U}_\varphi|\psi_m^2\rangle$ using Supplementary Equation (14) with its output probabilities. The CRB can be obtained by numerically minimizing the $\mathrm{Tr}[\mathbf{F}_C^{-1}(\boldsymbol{\varphi})]$, and they are also shown in Supplementary Table I with varying d from 1 to 5.

SUPPLEMENTARY NOTE 3 - ANALYSIS OF 4-MODE 2002 STATE

A. Analysis on the ideal probe state and the ideal quarter

We start from the ideal multi-mode $N00N$ state with $m=4$ and $N=2$ having the following form,

$$|\psi_4^2\rangle = \frac{1}{2\sqrt{2}}(\hat{a}_0^\dagger\hat{a}_0^\dagger + \hat{a}_1^\dagger\hat{a}_1^\dagger + \hat{a}_2^\dagger\hat{a}_2^\dagger + \hat{a}_3^\dagger\hat{a}_3^\dagger)|0\rangle = \frac{1}{2}(|2000\rangle + |0200\rangle + |0020\rangle + |0002\rangle). \tag{16}$$

The phase shifts $\mathbf{U}_\varphi$ which a 4-mode 2002 input probe state $|\psi_4^2\rangle$ undergoes and the ideal quarter $\mathbf{U}_q$ are given by

$$\mathbf{U}_\varphi = \begin{pmatrix} 1 & 0 & 0 & 0 \\ 0 & e^{i\varphi_1} & 0 & 0 \\ 0 & 0 & e^{i\varphi_2} & 0 \\ 0 & 0 & 0 & e^{i\varphi_3} \end{pmatrix}, \text{and } \mathbf{U}_q = \frac{1}{2}\begin{pmatrix} 1 & 1 & 1 & 1 \\ 1 & e^{i\theta} & -1 & -e^{i\theta} \\ 1 & -1 & 1 & -1 \\ 1 & -e^{i\theta} & -1 & e^{i\theta} \end{pmatrix},$$

respectively. The free internal phase θ is determined by the experimental quarter [1] and here we set $\theta = 0$. After the state $|\psi_4^2\rangle$ undergoes phase shifts $\mathbf{U}_\varphi$, the phase encoded state $|\psi'_\varphi\rangle = \mathbf{U}_\varphi|\psi_4^2\rangle = \frac{1}{2}(|2000\rangle + e^{i2\varphi_1}|0200\rangle + e^{i2\varphi_2}|0020\rangle + e^{i2\varphi_3}|0002\rangle)$ has the information about φ_i $(i=1,2,3)$. Using the symmetric logarithmic derivative in Supplementary Equation (12) to the output state $|\psi'_\varphi\rangle$, we can obtain the QFIM as the following,

$$\mathbf{F}_Q = \begin{pmatrix} 3 & -1 & -1 \\ -1 & 3 & -1 \\ -1 & -1 & 3 \end{pmatrix}. \tag{17}$$

Then, the QCRB can be obtained by calculating $\mathrm{Tr}[\mathbf{F}_Q^{-1}]$= 1.5. Note that 1.5 is lower than the QCRB of $|\psi_{\mathrm{Fock}}\rangle$, 2.33.

The CFIM was also obtained by considering not only the phase encoding $\mathbf{U}_\varphi$, but also a detection scheme using a quarter $\mathbf{U}_q$. After the probe states $|\psi_4^2\rangle$ undergoes phase shifts and the quarter transformation, the output state $|\psi'_{\mathrm{out}}\rangle = \mathbf{U}_q\mathbf{U}_\varphi|\psi_4^2\rangle$ has following form:

$$\begin{aligned} |\psi_{\mathrm{out}}\rangle = {} & c_0|2000\rangle + c_1|0200\rangle + c_2|0020\rangle + c_3|0002\rangle + c_4|1100\rangle + c_5|0011\rangle \\ & + c_6|1010\rangle + c_7|0101\rangle + c_8|1001\rangle + c_9|0110\rangle, \end{aligned} \tag{18}$$

where the detection probability is $P_l(\boldsymbol{\varphi}) = |c_l|^2$ with satisfying the normalization condition $\sum_{l=0}^{9} P_l(\boldsymbol{\varphi}) = 1$, and we have the following probability set:

$$
\begin{aligned}
P_0 = P_1 = P_2 = P_3 &= \frac{1}{32}\left(\cos\left(2\varphi_1\right) + \cos\left(2\left(\varphi_1 - \varphi_2\right)\right) + \cos\left(2\varphi_2\right) + \cos\left(2\left(\varphi_1 - \varphi_3\right)\right) + \cos\left(2\left(\varphi_2 - \varphi_3\right)\right) + \cos\left(2\varphi_3\right) + 2\right), \\
P_4 = P_5 &= \frac{1}{16}\left(\cos\left(2\varphi_1\right) - \cos\left(2\left(\varphi_1 - \varphi_2\right)\right) - \cos\left(2\varphi_2\right) - \cos\left(2\left(\varphi_1 - \varphi_3\right)\right) + \cos\left(2\left(\varphi_2 - \varphi_3\right)\right) - \cos\left(2\varphi_3\right) + 2\right), \\
P_6 = P_7 &= \frac{1}{16}\left(-\cos\left(2\varphi_1\right) - \cos\left(2\left(\varphi_1 - \varphi_2\right)\right) + \cos\left(2\varphi_2\right) + \cos\left(2\left(\varphi_1 - \varphi_3\right)\right) - \cos\left(2\left(\varphi_2 - \varphi_3\right)\right) - \cos\left(2\varphi_3\right) + 2\right), \\
P_8 = P_9 &= \frac{1}{16}\left(-\cos\left(2\varphi_1\right) + \cos\left(2\left(\varphi_1 - \varphi_2\right)\right) - \cos\left(2\varphi_2\right) - \cos\left(2\left(\varphi_1 - \varphi_3\right)\right) - \cos\left(2\left(\varphi_2 - \varphi_3\right)\right) + \cos\left(2\varphi_3\right) + 2\right).
\end{aligned}
\tag{19}
$$

The theoretical fittings on the experimental data of two-photon coincidence in Figure 3**d-f** in main text are based on the above $P_l(\boldsymbol{\varphi})$ results, i.e. $P_0 = \mathrm{C}_{b_{0'} b_{0''}}$, $P_4 = \mathrm{C}_{b_{0'} b_1}$, $P_6 = \mathrm{C}_{b_{0'} b_2}$, and $P_8 = \mathrm{C}_{b_{0'} b_3}$.

We can evaluate the CFIM as Supplementary Equation (14) using the $P_l(\boldsymbol{\varphi})$ set. Then, we numerically minimize the $\mathrm{Tr}[\mathbf{F}_C^{-1}(\boldsymbol{\varphi})]$ by changing the phase shift of φ_1, φ_2, and φ_3. We find the minimum values of $\mathrm{Tr}[\mathbf{F}_C^{-1}(\boldsymbol{\varphi})]= 1.5$ when $\varphi_1 \simeq \pi/2$, $\varphi_2 = 0$, and $\varphi_3 = \pi/2$. Note that $\mathrm{Tr}[\mathbf{F}_C^{-1}(\boldsymbol{\varphi})]= 1.5$ has the same value of $\mathrm{Tr}[\mathbf{F}_Q^{-1}]$, meaning that the CRB can saturate the QCRB value using an ideal quarter. By substituting $\varphi_2 = 0$ and $\varphi_3 = \pi/2$ into Supplementary Equation (19), we can obtain the probabilities as a function of φ_1 with $P_0 = P_1 = P_2 = P_3 = \cos^2(\varphi_1)/16$, $P_4 = P_5 = \cos^2(\varphi_1)/8$, $P_6 = P_7 = [5 - 3\cos(2\varphi_1)]/16$, and $P_8 = P_9 = \cos^2(\varphi_1)/8$, and they are used for obtain the CFIM. Then, the CFIM at $\varphi_2 = 0$ and $\varphi_3 = \pi/2$ can be obtained using Supplementary Equation (14):

$$
\mathbf{F}_C(\boldsymbol{\varphi}) = \begin{pmatrix}
\frac{-24\sin^2(\varphi_1)}{3\cos(2\varphi_1)-5} & \frac{8\sin^2(\varphi_1)}{3\cos(2\varphi_1)-5} & \frac{8\sin^2(\varphi_1)}{3\cos(2\varphi_1)-5} \\
\frac{8\sin^2(\varphi_1)}{3\cos(2\varphi_1)-5} & \frac{8\sin^2(\varphi_1)(\cos(2\varphi_1)-2)}{3\cos(2\varphi_1)-5} & \frac{8\sin^4(\varphi_1)}{3\cos(2\varphi_1)-5} \\
\frac{8\sin^2(\varphi_1)}{3\cos(2\varphi_1)-5} & \frac{8\sin^4(\varphi_1)}{3\cos(2\varphi_1)-5} & \frac{8\sin^2(\varphi_1)(\cos(2\varphi_1)-2)}{3\cos(2\varphi_1)-5}
\end{pmatrix}.
\tag{20}
$$

Note that the CFIM $\mathbf{F}_C(\boldsymbol{\varphi})$ can have the same value of $\mathbf{F}_Q$ when $\varphi_1 \cong \pi/2$, $\varphi_2 = 0$ and $\varphi_3 = \pi/2$ as given in the following:

$$
\mathbf{F}_C(\boldsymbol{\varphi}) = \mathbf{F}_Q = \begin{pmatrix} 3 & -1 & -1 \\ -1 & 3 & -1 \\ -1 & -1 & 3 \end{pmatrix}.
\tag{21}
$$

Then we can calculate the CRB with $\mathrm{Tr}[\mathbf{F}_C^{-1}(\boldsymbol{\varphi})]= 1.5$, and it saturates the QCRB with $\mathrm{Tr}[\mathbf{F}_Q^{-1}]$=$\mathrm{Tr}[\mathbf{F}_C^{-1}(\boldsymbol{\varphi})]= 1.5$ at $\varphi_1 \cong \pi/2$ $\varphi_2 = 0$ and $\varphi_3 = \pi/2$.

B. Analysis considering experimental errors

By considering the experimental imperfection, we start from the Bells state of $|\Phi^+\rangle$ as described first line of Eq.(3) in main text. Here, we consider the noise from experimental imperfection as follow [5]:

$$
|\Phi^+_{\mathrm{exp}}\rangle = \epsilon \frac{1}{\sqrt{2}}(\hat{\alpha}_0^{H\dagger}\hat{\alpha}_1^{H\dagger} + \hat{\alpha}_0^{V\dagger}\hat{\alpha}_1^{V\dagger})|0\rangle + \sqrt{1-|\epsilon|^2}\frac{1}{\sqrt{2}}(\hat{\alpha}_0^{\prime H\dagger}\hat{\alpha}_1^{H\dagger} + \hat{\alpha}_0^{\prime V\dagger}\hat{\alpha}_1^{V\dagger})|0\rangle
\tag{22}
$$

where $\hat{\alpha}_j^{H\dagger}$ ($\hat{\alpha}_j^{V\dagger}$) creates a photon at α_j with a horizontal (vertical) polarization shown in Supplementary Figure 7, and $\hat{\alpha}_j^{\prime H\dagger}$ ($\hat{\alpha}_j^{\prime V\dagger}$) creates an distinguishable photon at same position due to experimental imperfection. Note that photons created from $\hat{\alpha}_j^{H\dagger}$ ($\hat{\alpha}_j^{V\dagger}$) and $\hat{\alpha}_j^{\prime H\dagger}$ ($\hat{\alpha}_j^{\prime V\dagger}$) are distinguishable and they do not interfere each other. ϵ is real parameter and varying from 0 to 1. Then we represented beam splitter transformation at lateral beam splitter as follow:

$$
\hat{\alpha}_j^{H\dagger} \to \frac{\hat{\beta}_{j+1}^{H\dagger} - i\hat{\beta}_j^{H\dagger}}{\sqrt{2}}, \hat{\alpha}_j^{V\dagger} \to \frac{\hat{\beta}_{j+1}^{V\dagger} - i\hat{\beta}_j^{V\dagger}}{\sqrt{2}}, \hat{\alpha}_j^{\prime H\dagger} \to \frac{\hat{\beta}_{j+1}^{\prime H\dagger} - i\hat{\beta}_j^{\prime H\dagger}}{\sqrt{2}}, \hat{\alpha}_j^{\prime V\dagger} \to \frac{\hat{\beta}_{j+1}^{\prime V\dagger} - i\hat{\beta}_j^{\prime V\dagger}}{\sqrt{2}}
\tag{23}
$$

where $\hat{\beta}_j^{H\dagger}$ ($\hat{\beta}_j^{V\dagger}$) creates a photon at β_j with horizontal (vertical) polarization, and also $\hat{\beta}_j^{H\dagger}$ ($\hat{\beta}_j^{V\dagger}$) is distinguishable with $\hat{\beta}_j^{\prime H\dagger}$ ($\hat{\beta}_j^{\prime V\dagger}$) and they do not interfere each other ($j \equiv \bmod 2$). After transformation at PBS and HWP at 45°,

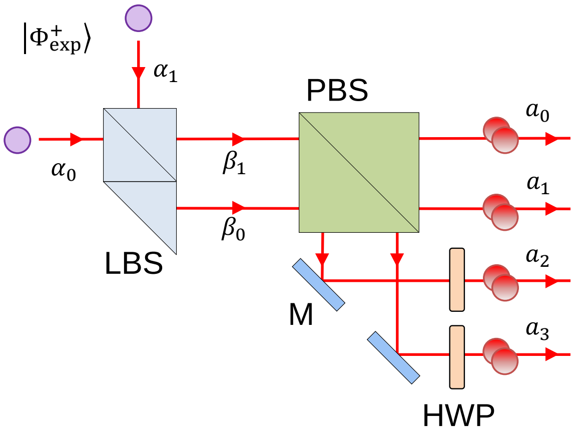

Supplementary Figure 7. **Generation of $|\psi_4^2\rangle_{\rm exp}$ state.** The $|\psi_4^2\rangle_{\rm exp}$ state is generated from Bell state of $|\Phi^+_{\rm exp}\rangle$ including experimental errors.

we can define the $\hat{a}_j^\dagger$ as follow:

$$\hat{\beta}_1^{H\dagger} \to \hat{a}_0^\dagger, \hat{\beta}_0^{H\dagger} \to \hat{a}_1^\dagger, \hat{\beta}_1^{V\dagger} \to \hat{a}_2^\dagger, \hat{\beta}_0^{V\dagger} \to \hat{a}_3^\dagger \tag{24}$$

where $\hat{a}_j^\dagger$ create a photon at a_j and similarly for distinguishable photon a'_j. Here, a_j are used for input state at quarter. Finally, the noise considered 4-mode 2002 state becomes as follow:

$$|\psi_4^2\rangle_{\rm exp} = \epsilon\frac{1}{2\sqrt{2}}(\hat{a}_0^\dagger\hat{a}_0^\dagger+\hat{a}_1^\dagger\hat{a}_1^\dagger+\hat{a}_2^\dagger\hat{a}_2^\dagger+\hat{a}_3^\dagger\hat{a}_3^\dagger)|0\rangle+\sqrt{1-|\epsilon|^2}\frac{1}{2\sqrt{2}}(\hat{a}_0^\dagger\hat{a}_0'^\dagger+\hat{a}_1^\dagger\hat{a}_1'^\dagger+\hat{a}_2^\dagger\hat{a}_2'^\dagger+\hat{a}_3^\dagger\hat{a}_3'^\dagger+i(\hat{a}_0'^\dagger\hat{a}_1^\dagger+\hat{a}_0^\dagger\hat{a}_1'^\dagger+\hat{a}_2'^\dagger\hat{a}_3^\dagger+\hat{a}_2^\dagger\hat{a}_3'^\dagger))|0\rangle \tag{25}$$

Then $|\psi_4^2\rangle_{\rm exp}$ undergoes the phase shift and experimental quarter to be $|\psi_{\rm out}\rangle_{\rm exp} = \mathbf{U}_{\rm q,exp}\mathbf{U}_\varphi|\psi_4^2\rangle_{\rm exp}$. Here, we could calculate the fitting function of $P_l^{\rm exp}(\boldsymbol{\varphi})$ for $l = 0, 1, ..., 9$ from the $|\psi_{\rm out}\rangle_{\rm exp}$ following the similar calculation described in A. Analysis on the ideal probe state and the ideal quarter.

$$\begin{aligned}|\psi_{\rm out}\rangle_{\rm exp} = c_0^{\rm exp}|2000\rangle + c_1^{\rm exp}|0200\rangle + c_2^{\rm exp}|0020\rangle + c_3^{\rm exp}|0002\rangle + c_4^{\rm exp}|1100\rangle + c_5^{\rm exp}|0011\rangle \\ + c_6^{\rm exp}|1010\rangle + c_7^{\rm exp}|0101\rangle + c_8^{\rm exp}|1001\rangle + c_9^{\rm exp}|0110\rangle\end{aligned} \tag{26}$$

Note that $c_l^{\rm exp}$ is function of unknown phases $\boldsymbol{\varphi}$ and error parameter ϵ, then detection probabilities are $|c_l^{\rm exp}|^2 = P_l^{\rm exp}(\boldsymbol{\varphi})$. In our experiment, we measured the post-selected two-photon coincidence counts, C_i, ($i = b_{0'}b_{0''}, b_{0'}b_1, b_{0'}b_2$, and $b_{0'}b_3$), and to obtain the probability sets we normalized coincidence counts with following relations $P_0^{\rm exp} = P_1^{\rm exp} = P_2^{\rm exp} = P_3^{\rm exp} = P_{b_0b_0}^{\rm m}$, $P_4^{\rm exp} = P_5^{\rm exp} = P_{b_0b_1}^{\rm m}$, $P_6^{\rm exp} = P_7^{\rm exp} = P_{b_0b_2}^{\rm m}$, and $P_8^{\rm exp} = P_9^{\rm exp} = P_{b_0b_3}^{\rm m}$ by assuming that experimentally reconstructed quarter is almost same with an ideal quarter with fidelity of $|\mathrm{Tr}[\mathbf{U}_{q,\rm exp}(\mathbf{U}_q)^\dagger]|/4 = 0.991$. Note that, for ideal quarter, following relations are always satisfied; $P_0 = P_1 = P_2 = P_3$, $P_4 = P_5$, $P_6 = P_7$, and $P_8 = P_9$. For example, $P_{b_0b_0}^{\rm m}$ are obtained by $P_{b_0b_0}^{\rm m} = \mathrm{C}_{b_{0'}b_{0''}}/(4\mathrm{C}_{b_{0'}b_{0''}} + 2\mathrm{C}_{b_{0'}b_{0''}} + 2\mathrm{C}_{b_{0'}b_{0''}} + 2\mathrm{C}_{b_{0'}b_{0''}})$. Then $P_0^{\rm exp}$, $P_4^{\rm exp}$, $P_6^{\rm exp}$, and $P_8^{\rm exp}$ are used as a fitting function by changing the $\boldsymbol{\varphi}$ and ϵ for experimentally obtained detection probabilities of $P_{b_0b_0}^{\rm m}$, $P_{b_0b_1}^{\rm m}$, $P_{b_0b_2}^{\rm m}$, and $P_{b_0b_3}^{\rm m}$ as shown in Figure 4**a** in main text. The probabilities of $P_l^{\rm exp}(\boldsymbol{\varphi})$ are function of unknown phases $\boldsymbol{\varphi}$ and noise parameter ϵ, and they are used for experimental fitting functions of Figure 4 in main text. The interference fringes in Figure 4**a** in main text are fitted by $P_l^{\rm exp}(\boldsymbol{\varphi})$ obtained from $|\psi_{\rm out}\rangle_{\rm exp}$ with fitting parameters of $\varphi_2 = (-0.07 \pm 0.01)\pi$, $\varphi_3 = (0.52 \pm 0.01)\pi$, and $\epsilon = 0.956 \pm 0.007$. Then CFIM $\mathbf{F}_C^{\rm exp}(\boldsymbol{\varphi})$ can be evaluated using Supplementary Equation (14) from $P_l^{\rm exp}(\boldsymbol{\varphi})$ with fitting parameters, and we numerically minimize the $\mathrm{Tr}[(\mathbf{F}_C^{\rm exp})^{-1}(\boldsymbol{\varphi})]$ by changing the φ_1 to obtain the minimum CRB. We can calculate the CFIM $\mathbf{F}_C^{\rm exp}(\boldsymbol{\varphi})$ which has the minimum CRB at $\varphi_1 \simeq 0.47\ \pi$, $\varphi_2 = -0.07\ \pi$, and $\varphi_3 = 0.52\ \pi$ as follows:

$$\mathbf{F}_C^{\rm exp}(\boldsymbol{\varphi}) = \begin{pmatrix} 2.33 & -0.63 & -0.93 \\ -0.63 & 2.70 & -1.09 \\ -0.93 & -1.09 & 2.66 \end{pmatrix}. \tag{27}$$

Then we can evaluate the CRB by calculating the $\mathrm{Tr}[(\mathbf{F}_C^{\rm exp})^{-1}(\boldsymbol{\varphi})]$= 1.85 ± 0.01. The errors are calculated from one standard deviation of the fitting parameter ϵ. We also analyse the impact of normalization assumption. The

CRB value are obtained to be 1.879 using $|\psi_{\rm out}\rangle_{\rm exp}$ with fitting parameters of $\varphi_2 = -0.07\ \pi$, $\varphi_3 = 0.52\ \pi$, and $\epsilon = 0.956 \pm 0.007$ without normalization assumption.

In addition, we analyse the diagonal term of the CFIM and the corresponding CRB depending on φ_1, φ_2, and φ_3 using the experimentally obtained fitting function with $\varphi_1 \simeq 0.47\ \pi$, $\varphi_2 = -0.07\ \pi$ $\varphi_3 = 0.52\ \pi$, and $\epsilon = 0.956$. We also compared the diagonal term of the CFIM and the CRB using $P_l(\boldsymbol{\varphi})$ from an ideal 2002 state and an ideal quarter when $\varphi_1 \simeq 0.5\ \pi$, $\varphi_2 = 0$ $\varphi_3 = 0.5\ \pi$, and $\epsilon = 1$. Supplementary Figure 8 presents the results. At first, a diagonal term of the CFIM from an ideal analysis are obtained as a function of φ_j when the other phases are fixed, for example, when φ_1 is scanned, both φ_2 and φ_3 are fixed at 0 and 0.5 π, respectively, and similarly for scanning either φ_2 or φ_3. We find that the two diagonal terms are always the same, for example, $F_{C(2,2)}$ and $F_{C(3,3)}$ are exactly the same and they are overlapped in Supplementary Figure 8**a**. The experimental CFIM curves are also obtained by using the experimentally obtained fitting function of $P_l^{\rm exp}(\boldsymbol{\varphi})$ and they are shown in Supplementary Figure 8**d-f**. The riffles near the local maximum points are originated from the slight shift of the point where the derivative of experimentally obtained $P_l^{\rm exp}(\boldsymbol{\varphi})$ is 0 (See Figure4**a** in main text). The corresponding CRB from ideal and experimental analyses are shown in Supplementary Figure 8**g-i**.

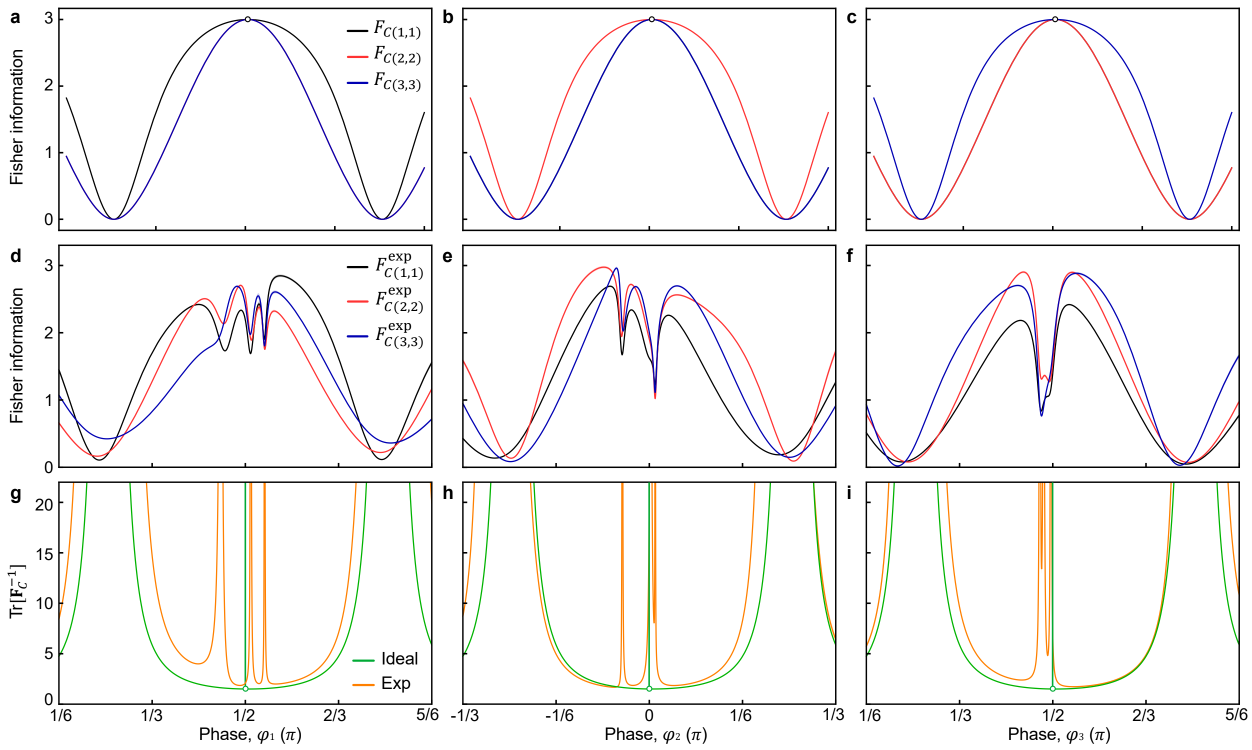

Supplementary Figure 8. **Fisher information and corresponding Cramer-Rao bound for ideal and experimental 2002 states. a-c,** Classical Fisher information of $F_{C(1,1)}$, $F_{C(2,2)}$, and $F_{C(3,3)}$ with an ideal 2002 state and an ideal quarter. **d-f,** Classical Fisher information of $F^{\rm exp}_{C(1,1)}$, $F^{\rm exp}_{C(2,2)}$, and $F^{\rm exp}_{C(3,3)}$ with $|\psi_4^2\rangle_{\rm exp}$ and the experimental quarter $\mathbf{U}_{q,\rm exp}$. **g-i,** Corresponding Cramer-Rao bound obtained from **a-c** ideal calculation and **d-f** experimental analysis.

SUPPLEMENTARY NOTE 4 - ANALYSIS WITH PHOTONIC LOSSES

Our scheme, shown in Figure 2**c** in main text, includes the following major losses: 1) collection efficiency of Bell state η_b, 2) optical loss at quarter η_q, 3) collection efficiency before quarter η_c, 4) detection effeiciency of superconducting nanowire single photon detctor (SNSPD) η_d, and 5) the other losses from optical components $\eta_{\rm etc}$. We analyse the performance of our work by considering the all losses with the CRB values. We start from the ideal 2002 input state with Supplementary Equation (16),

$$|\psi_4^2\rangle = \frac{1}{2\sqrt{2}}(\hat{a}_0^\dagger\hat{a}_0^\dagger + \hat{a}_1^\dagger\hat{a}_1^\dagger + \hat{a}_2^\dagger\hat{a}_2^\dagger + \hat{a}_3^\dagger\hat{a}_3^\dagger)|0\rangle. \tag{28}$$

Efficiency	Ours	State-of-art efficiency
η_b	0.76	0.95 [7]
η_q	0.92	0.92
η_c	0.6	0.95 [7]
η_d	0.8	0.95 [8, 9]
η_{etc}	0.95	1
η_{tot}	0.32	0.79

TABLE II. Experimental efficiencies of our setup and the best efficiencies of previous work, to the our best knowledge.

and the losses are modeled as $\hat{a}_j^\dagger \rightarrow \sqrt{\eta_b\eta_q\eta_c\eta_d\eta_{\text{etc}}}\hat{a}_j^\dagger$ considering all losses [6]. Then loss considered input state becomes $|\psi_4^2\rangle_{\text{loss}} = \eta_{\text{tot}}|\psi_4^2\rangle$ where $\eta_{\text{tot}} = \eta_b\eta_q\eta_c\eta_d\eta_{\text{etc}}$. After $|\psi_4^2\rangle_{\text{loss}}$ state undergoes phase shift operation and quarter transformation, then the final state becomes $|\psi_{\text{out}}\rangle_{\text{loss}} = \mathbf{U}_q\mathbf{U}_\varphi|\psi_4^2\rangle_{\text{loss}}$. Then we could obtained the loss considered probability set $P_l^{\text{loss}}(\boldsymbol{\varphi}) = \eta_{\text{tot}}^2 P_l(\boldsymbol{\varphi})$, which has the form of multiplied η_{tot}^2 to Supplementary Equation (19). Then we analyse the CRB values using loss considered probability set P_l^{loss}. Then we can find that the CRB becomes CRB$/\eta_{\text{tot}}^2$. In our experiments, the ideal CRB value is 1.5 and the loss considered CRB becomes $1.5/\eta_{\text{tot}}^2$. By replacing the all losses with values shown in Supplementary Table II, the ideal CRB becomes 14.68 and our experimental CRB 1.85 becomes 18.11. Note that the loss considered CRB values for $|\alpha\rangle$ and $|\psi_{\text{Fock}}\rangle$ becomes 29.37 and 23.93, respectively. Further, we analyze the CRB values with the help of state-of-art high efficiency of $\eta_b = \eta_c = 0.95$, $\eta_d = 0.95$ and $\eta_{\text{etc}} = 1$ achieved in previous works [7–9], the CRB values becomes $1.5/\eta_{\text{tot}}^2 = 2.40$ and $1.85/\eta_{\text{tot}}^2 = 2.97$. Note that the ideal CRB value from classical coherent states is 3. It indicates that our scheme can unconditionally violate the classical limit of 3 with the help of high-efficiency detector and optimized low-loss optical components.

[1] Pryde, G. J., & White, A. G. Creation of maximally entangled photon-number states using optical fiber multiports. *Phys. Rev. A* **68**, 052315 (2003).

[2] Liu, J., Yuan, H., Lu, X. M., & Wang, X. Quantum Fisher information matrix and multiparameter estimation. *J. Phys. A: Math. Theor.* **53**, 023001 (2019).

[3] Peruzzo, A., Laing, A., Politi, A., Rudolph, T., & O'Brien, J. L. Multimode quantum interference of photons in multiport integrated devices. *Nat. Commun.* **2**, 224 (2011).

[4] Kim, I. et al. Implementation of a 3 x 3 directionally-unbiased linear optical multiport. Preprint at https://arxiv.org/abs/2106.13473 (2021).

[5] Lyons, A., Knee, G. C., Bolduc, E., Roger, T., Leach, J., Gauger, E. M., & Faccio, D. Attosecond-resolution Hong-Ou-Mandel interferometry. *Sci. Advances* **4**, eaap9416 (2018).

[6] Datta, A., Zhang, L., Thomas-Peter, N., Dorner, U., Smith, B. J., & Walmsley, I. A. Quantum metrology with imperfect states and detectors. *Phys. Rev. A* **83**, 063836 (2011).

[7] Weston, M. M. et al. Efficient and pure femtosecond-pulse-length source of polarization-entangled photons. *Opt. Express* **24**, 10869-10879 (2016).

[8] Slussarenko, S., Weston, M. M., Chrzanowski, H. M., Shalm, L. K., Verma, V. B., Nam, S. W., & Pryde, G. J. Unconditional violation of the shot-noise limit in photonic quantum metrology. *Nat. Photon.* **11**, 700–703 (2017).

[9] You, C. et al. Multiphoton quantum metrology with neither pre-nor post-selected measurements. Preprint at https://arxiv.org/abs/2011.02454 (2020).
